# The CckA-ChpT-CtrA Phosphorelay System Is Regulated by Quorum Sensing and Controls Flagellar Motility in the Marine Sponge Symbiont *Ruegeria* sp. KLH11

**DOI:** 10.1371/journal.pone.0066346

**Published:** 2013-06-25

**Authors:** Jindong Zan, Jason E. Heindl, Yue Liu, Clay Fuqua, Russell T. Hill

**Affiliations:** 1 Institute of Marine and Environmental Technology, University of Maryland Center for Environmental Science, Baltimore, Maryland, United States of America; 2 Department of Biology, Indiana University, Bloomington, Indiana, United States of America; National Institutes of Health, United States of America

## Abstract

Bacteria respond to their environment via signal transduction pathways, often two-component type systems that function through phosphotransfer to control expression of specific genes. Phosphorelays are derived from two-component systems but are comprised of additional components. The essential *cckA-chpT-ctrA* phosphorelay in *Caulobacter crescentus* has been well studied and is important in orchestrating the cell cycle, polar development and flagellar biogenesis. Although *cckA, chpT* and *ctrA* homologues are widespread among the *Alphaproteobacteria*, relatively few is known about their function in the large and ecologically significant *Roseobacter* clade of the *Rhodobacterales*. In this study the *cckA-chpT-ctrA* system of the marine sponge symbiont *Ruegeria* sp. KLH11 was investigated. Our results reveal that the *cckA, chpT* and *ctrA* genes positively control flagellar biosynthesis. In contrast to *C. crescentus*, the *cckA*, *chpT* and *ctrA* genes in *Ruegeria* sp. KLH11 are non-essential and do not affect bacterial growth. Gene fusion and transcript analyses provide evidence for *ctrA* autoregulation and the control of motility-related genes. In KLH11, flagellar motility is controlled by the SsaRI system and acylhomoserine lactone (AHL) quorum sensing. SsaR and long chain AHLs are required for *cckA*, *chpT* and *ctrA* gene expression, providing a regulatory link between flagellar locomotion and population density in KLH11.

## Introduction

Roseobacters represent an abundant and important marine bacterial group in the *Alphaproteobacteria*. Members from this group can mediate key biogeochemical processes and account for up to 30% of bacterioplankton cells in some coastal environments [1]. Many roseobacters have been experimentally shown to exhibit flagellar motility, an important trait for their physical associations with eukaryotic cell surfaces or organic particles [2]. For example, in *Silicibacter* sp. TM1040 flagellar mutants are defective in attaching to and forming biofilms on the dinoflagellates with which this bacterium is associated [3]. *Alphaproteobacteria*, including roseobacters are also found in association with marine sponges [4], [5]. *Ruegeria* sp. KLH11 is a sponge symbiont within the roseobacterial *Silicibacter*-*Ruegeria* subgroup which is consistently and specifically isolated from soft-bodied marine sponges such as species of *Mycale* and *Ircinia* [6]. KLH11 has been developed as a model for studying the interactions of bacteria with sponge hosts.

Two-component system signal transduction pathways, comprised of two or more proteins, are some of the most prevalent means by which bacteria sense, respond, and adapt to changes in their environment or their intracellular state. In their simplest form two-component systems consist of a sensor histidine kinase and a cognate response regulator, through which phosphotransfer controls the regulatory output [7]. More complex systems can have multiple components, and individual regulators can have multiple phosphotransfer activities. In the alphaproteobacterial developmental model system *Caulobacter crescentus* the response regulator CtrA acts to control the cell cycle and is essential for viability. CtrA is phosphorylated on a conserved asparate residue (D51), via a phosphorelay pathway that initiates with the histidine kinase CckA. When active, CckA undergoes an intramolecular phosphotransfer between a conserved histidine and an aspartate in its receiver domain at the carboxy terminal end of the protein [7]. Active CckA subsequently phosphorylates the ChpT histidine phosphotransferase (Hpt). ChpT can transfer phosphate to either of two response regulators, CpdR or CtrA [8], [9]. CpdR normally inhibits CtrA, but is inactive when it is phosphorylated. Phospho-CtrA, relieved of CpdR inhibition, is an active transcriptional regulator that controls about 26% (144/553) of the genes involved in cell cycle progression and also controls flagellar motility in *C. crescentus* [10]. Members of the *Rhodobacterales* such as KLH11 encode *cckA*, *chpT*, and *ctrA* homologues [11], but generally no *cpdR* homologue [12]. In *Silicibacter* sp. TM1040, a relative of *Ruegeria* sp. KLH11, the *cckA*, *chpT* (referred as *flaB*) and *ctrA* genes are required for flagellar motility, but in contrast to *C. crescentus* these genes are non-essential [13].

Bacterial flagellar motility displays a critical role in many microbial processes, such as chemotaxis, colonization of hosts, and biofilm formation [14]. The biosynthesis of flagella is an ordered process that requires the coordinated and temporal regulation of many different genes via a very complex regulatory hierarchy. For bacteria in which flagellar assembly has been well studied there is generally a primary regulator that initiates expression of the flagellar gene hierarchy and is referred as the master regulator. Several different types of master regulators, including CtrA from *C. crescentus*, have been identified. FlhDC is the most extensively studied master regulator in both *Escherichia coli* and *Salmonella typhimurium* [15], [16]. FleQ and FlrA are the master regulators in *Pseudomonas aeruginosa* and *Vibrio cholerae* [17], [18], respectively. Although flagellar motility is common among the roseobacters [2], little is known about its regulation.

We recently reported that the sponge symbiont *Ruegeria* sp. KLH11 utilizes two distinct but interconnected quorum sensing (QS) systems, with the LuxR-LuxI homologues SsaRI and SsbRI, that rely upon an overlapping set of long chain acylhomoserine lactone (AHL) signal molecules [19]. Many bacteria use intercellular signals such as AHLs to monitor their population density and accordingly regulate the expression of specific gene sets in crowded conditions. The SsaRI system is required for flagellar assembly and flagellar gene expression in KLH11 whereas the SsbRI system has no influence on motility. KLH11 specifically switches into a motile phase at high population densities, and this requires SsaRI [19]. Although it is possible that SsaR functions as the primary regulator of motility, it is more likely that it controls expression of a downstream regulator specific for the flagellar genes. For example, in *Burkholderia glumae* the *tofRI* QS system regulates the expression of *flhDC*, which in turn directly controls motility [20]. Although the FlhDC and FleQ/FlrA homologues are not present in *Ruegeria pomeroyi* DSS-3 or in KLH11 genome sequences [11], [21], it is possible that the *cckA-chpT*-*ctrA* pathway acts in this capacity. In this study we examined whether 1) *cckA*, *chpT* and *ctrA* genes are essential for the viability of KLH11; 2) they can control flagellar motility; 3) they are influenced by the SsaRI system. Our results show clearly that *cckA*, *chpT* and *ctrA* are non-essential, are tightly regulated by QS, and act downstream of QS in controlling flagellar motility.

## Materials and Methods

### Strains, growth conditions and plasmid transformation

Bacterial strains and plasmids used in this study are listed in Table S1. *Ruegeria* sp. KLH11 and KLH11-EC1 derivatives were grown in Marine Broth 2216 (MB2216) (BD, Franklin Lakes, NJ) at 28°C. *Escherichia coli* strains were grown at 37°C in Luria-Bertani (LB) broth (10 g·L^−1^ tryptone, 5 g·L^−1^ yeast extract, 10 g·L^−1^ NaCl). *Agrobacterium tumefaciens* strains were grown in AT minimal medium supplemented with 0.5% glucose and 15 mM (NH_4_)_2_SO_4_ (ATGN) [22]. Antibiotics were used at the following concentrations (µg·mL^−1^): (i) *E. coli* (gentamicin, Gm, 25; kanamycin, Km, 50; spectinomycin, Sp, 100), (ii) KLH11 (Km, 100; rifampicin, Rif, 200; Gm, 25; Sp, 100) (iii), *A. tumefaciens* (Gm, 300; Sp, 200).

Plasmids were introduced into KLH11 and derivatives using either electroporation or conjugation [19] and into *E. coli* using standard methods of chemical transformation and into *A. tumefaciens* using a standard electroporation method [23].

### Deletion of ssaR and generation of *cckA*, *chpT*, and *ctrA* null mutants

DNA manipulations were performed using standard techniques or per manufacturers' specifications [24]. Restriction enzymes and Phusion™ High-Fidelity DNA Polymerase were obtained from New England Biolabs (Ipswich, MA). Oligonucleotides, listed in Table S2, were obtained from Integrated DNA Technologies (Coralville, IA). DNA sequencing was performed on an ABI3700 automated sequencer by the BioAnalytical Services Laboratory at the Institute of Marine and Environmental Technology (Baltimore, MD). To generate an in-frame, markerless deletion of the *ssaR* gene, splicing by overlap extension (SOE) polymerase chain reaction (PCR) was used [25]. An approximately 500-bp fragment upstream of and including the first three codons of the *ssaR* coding sequence was amplified using primers ssaR D1 and ssaR D2. An approximately 500-bp fragment downstream of and including the *ssaR* stop codon was amplified using primers ssaR D3 and ssaR D4. Primers ssaR D2 and ssaR D3 were designed to contain an 18 bp complementary sequence at the 5′ end (the “overlap”) to facilitate the SOEing reaction [26]. Following initial amplification the two fragments were gel purified and used as template in a second round of PCR with primers ssaR D1 and ssaR D4 generating an approximately 1 kb SOE fragment containing a fusion of the upstream and downstream regions of the *ssaR* locus. Primers ssaR D1 and ssaR D4 were designed to allow direct cloning using the In-Fusion Clone Kit. The final SOE fragment was gel purified and cloned into the *sacB* counter-selectable vector pNPTS138 that had been previously digested with *Eco*RI. The resulting plasmid, pJZ014, was confirmed by sequencing and conjugated into *Ruegeria* sp. KLH11-EC1 (Rif^R^). The spontaneous Rif^R^ KLH11 strain was used to provide counter-selection against the *E. coli* donors throughout this study. The suicide vector pNPTS138 is a ColE1 plasmid carrying kanamycin resistance and is unable to replicate in *Ruegeria* sp. KLH11 [19]. Transconjugants were plated onto Marine Agar 2216 (MA2216) (BD, Franklin Lakes, NJ) plates supplemented with both Rif and Km to select for Rif^R^ Km^R^ plasmid integrants. Presumptive integrants were tested for sucrose sensitivity, verifying introduction of the *sacB* counter-selectable marker on pNPTS138, by plating on MA2216 plates supplemented with Rif, Km, and 5% (w/v) sucrose. Rif^R^ Km^R^ Suc^S^ colonies were subcultured in MB2216 without Km and plated on 5% sucrose MA2216 plates without Km to select for Suc^R^ allelic replacement candidates. Candidates were verified to be Km^S^ by patching onto MA2216 plates supplemented with Km. Deletion of the targeted *ssaR* locus was confirmed by PCR using primers ssaR D1 and ssaR D4 and the Δ*ssaR* strain was designated JZ03.

Null mutations in the *Ruegeria* sp. KLH11 *cckA*, *chpT*, and *ctrA* homologues were generated using Campbell-type recombinational mutagenesis. Internal gene fragments were generated by PCR using primers cckA P1/cckA P2, chpT P1/chpT P2, and ctrA P1/ctrA P2, respectively, using KLH11 genomic DNA as template. The partial *cckA*, *chpT*, and *ctrA* fragments were cloned directly into the pCR2.1-TOPO vector (Invitrogen, Grand Island, NY) and then subcloned into pVIK112, a suicide vector with an R6K conditional replication origin [27], creating plasmids pJZ003 (truncated at codon 513), pJZ004 (truncated at codon 160), and pJZ005 (truncated at codon 173), respectively. These plasmids were then conjugated into KLH11-EC1. Presumptive Km^R^ transconjugants were selected and confirmed by PCR amplification using the primer 3 designated for each of the three genes that is located upstream of the recombined fragments and the primer 112R that is located downstream of the *Kpn*I recognition site on the plasmid pVIK112 [27] and the amplicons were sequenced. The *cckA^−^*, *chpT^−^*, and *ctrA^−^* mutants were designated JZ04, JZ05, and JZ06, respectively. To create strains JZ07-JZ12 plasmids pJZ003, pJZ004, and pJZ005 were conjugated into Δ*ssaI* strain (SK01) and Δ*ssaR* strain (JZ03), respectively. The Km^R^ recombinants were selected and confirmed as for strains JZ04-JZ06.

A transcriptional fusion of *E. coli lacZ* immediately downstream of the KLH11 *cckA* homologue at its native genomic location was generated by PCR amplifying a 3′ fragment of the *cckA* gene, ending at the stop codon, using primers cckAintactF and cckAintactR. The PCR amplicon was cloned into pCR2.1-TOPO and then subcloned into pVIK112, creating pJZ012. This plasmid was conjugated into KLH11 and transconjugants were selected and confirmed as described above. Campbell-type recombination results in *lacZ* fused to the 3′ end of the native *cckA* locus, keeping the *cckA* gene-coding region intact.

### Cloning of phosphorelay components and promoter fusion constructs

Complementation constructs of *Ruegeria* sp. KLH11 homologues of *cckA* (pJZ006), *chpT* (pJZ007), and *ctrA* (pJZ008) were generated by PCR amplification of the coding regions of each gene using primers designated as P3 and P4 for each specific gene and KLH11 genomic DNA as template. An *E. coli lacZ* ribosomal binding site was engineered into the 5′ primer of each gene to allow for efficient translation. PCR products were cloned directly into the broad-host range vector pSRKGm that had been previously cut with SpeI [28] using the In-Fusion HD directional cloning system (Clontech, Mountain View, CA). The resulting expression plasmids carry each gene under the control of an isopropyl-β-d-1-thiogalactopyranoside (IPTG)-inducible *P_lac_* promoter. The insert carried by each construct was confirmed by sequencing.

The expression construct for the *A. tumefaciens cckA* homologue was created in the pSRKGm plasmid as described [29]. Expression constructs for the *A. tumefaciens chpT* and *ctrA* homologues were generated by PCR amplification with Phusion High-Fidelity DNA polymerase using purified wild-type *A. tumefaciens* C58 genomic DNA as template. Primers JEH48 and JEH53 were used to amplify the *chpT* locus and JEH50 and JEH54 were used for the *ctrA* locus (Table S2). Amplicons were cloned into vector pGEM-T Easy and sequenced. Each gene was then sub-cloned into pSRKGm using engineered *Nde*I and *Nhe*I restriction sites.

Fusions of the probable promoter regions for the KLH11 homologues of *cckA*, *chpT*, and *ctrA* to a promoterless *E. coli lacZ* β-galactosidase gene were created in plasmid pRA301 [30]. The intergenic region upstream of the *cckA* coding sequence was PCR amplified using primers cckA P5 and cckA P6. The upstream and downstream primers anneal 145 upstream, and 69 bp downstream, of the predicted *cckA* translational start site, respectively. The PCR product was fused with pCR2.1-TOPO and the insert was confirmed by DNA sequencing. The pCR2.1-TOPO derivative was digested with EcoRI and PstI, and the resulting fragment was ligated into similarly digested pRA301 creating pJZ009 which was confirmed by sequencing. Similarly, the intergenic regions upstream of the *chpT* and *ctrA* coding sequence were PCR amplified using primers chpT P5 and chpT P6 or ctrA P5 and ctrA P6, fused with pCR2.1-TOPO and then subcloned into pRA301, creating plasmids pJZ010 or pJZ011, respectively. Plasmids pEC112 (*P_lac_*-*ssaR*) and either pJZ009 (*P_cckA_*-*lacZ*), pJZ010 (*P_chpT_*-*lacZ*), or pJZ011 (*P_ctrA_*-*lacZ*) were electroporated into *A. tumefacien*s NTL4. Plasmids pBBR1-MCS5 [31] and either pJZ009, pJZ010 or pJZ011 were electroporated into *A. tumefacien*s NTL4 to serve as negative controls.

### Evaluation of flagellar-based motility and presence of flagella

Bacterial swimming motility assays were performed using MB2216 with 0.25% (w/v) agar supplemented with 200 µM IPTG to induce the *lac* promoter for complementation. Swim plates were inoculated with mid-log phase cultures of the relevant KLH11 strains using an inoculation needle. Plates were wrapped tightly with plastic film and incubated at 28°C. Swim ring diameters were measured and pictures taken after 8 days with a Nikon D90 digital camera.

Relative levels of flagellin in the wildtype, *cckA* ^−^, *chpT* ^−^, and *ctrA^−^* KLH11 strains were determined from culture supernatants followed by immunoblotting. Flagellin was enriched as described [32]. Strains were grown to mid-log phase in MB2216, supplemented with antibiotics when necessary, and then back-diluted to an OD_600_∼0.01 in 3 ml MB2216. Samples were collected at stationary phase and OD_600_ was measured. The samples were vigorously vortexed 30 sec and then centrifuged (5 min, 10,000× g) at 4°C. The resulting supernatant was transferred to a new centrifuge tube and polyethylene glycol was added to a final concentration of 2%. Following vortexing and 100 min incubation on ice, the mixtures were centrifuged (15 min, 17,400× g). The resulting precipitate was resuspended in 100 µl 1 X SDS lysis buffer and boiled at 100°C for 5–10 min. The denatured samples were separated on a 15% SDS-PAGE gel at 90 V for 4 h and then were transferred to a nitrocellulose membrane (Amersham Biosciences, Seattle, WA). Immunoblotting was performed with polyclonal antibody raised against whole flagella from *C. crescentus* (a gift from the laboratory of Y.V. Brun) at a dilution of 1∶20,000 as described by Zan et al. [19].

Staining of flagella on intact cells used a two-component stain modified from Mayfield and Inniss [33]. The first component contained equal volumes of saturated AlK(SO_4_)_2_·12H_2_O and 5% phenol in 10% tannic acid while the second component contained 12% crystal violet in 100% ethanol. Ten ml of a 10∶1 mixture of the two components was applied to the edge of a coverslip on a 3 ml wet mount for each strain. Flagella were observed within 5 min of staining on a Zeiss Axioskop 40 microscope equipped with an AxioCam MRm monochrome digital camera using a 100X oil immersion objective and bright field illumination.

### Quantification of phosphorelay component promoter activity

Promoter activities were quantified using *lacZ* translational and transcriptional fusions as indicated. β-galactosidase specific activity was measured as described previously, expressed in Miller Units, using o-nitrophenyl-β-D-galactopyranoside (ONPG) as substrate [19]. *Ruegeria* sp. KLH11 was grown in MB2216 supplemented with antibiotics as required overnight. Cultures were diluted approximately 100-fold to obtain an OD_600_∼0.01 in 3 ml MB2216 without antibiotics and incubated at 28°C. Mid-log phase KLH11 cultures were sampled and assayed for β-galactosidase activity immediately. Similarly, mid-log phase cultures of *A. tumefaciens* strain NTL4 were diluted at 1∶100 dilution to an OD_600_∼0.01 in 3 ml ATGN media and incubated at 28°C with shaking at 200 rpm to an OD_600_∼0.4. Mid-log phase cultures were measured for OD_600_ and frozen at −80°C and used for subsequent β-galactosidase assays. Exogenous AHL was added to each culture where indicated to a final concentration of 2 µM. 3-oxo-C16:1 Δ11cis-(l)-HSL was purchased from Cayman Chemical (Ann Arbor, Michigan). The A_420_ and OD_600_ were measured on a SpectrMax M5 microplate reader (Molecular Devices, Sunnyvale, CA) in 200 µl volume.

### Analysis of KLH11 CtrA-dependent gene expression

Expression of motility- and cell cycle-related genes was measured using qRT-PCR with specific primers (Table S2). KLH11 and derivatives were grown in MB2216 to stationary phase and 0.5 ml culture was collected and stored in 1 ml RNAprotect BacteriaReagent (Qiagen, Valencia, CA). The mixtures were centrifuged (10 min, 5100× g) and the cell pellets were stored at −80°C for subsequent RNA extraction. Total RNA was isolated using an RNeasy miniprep kit (Qiagen, Valencia, CA), with genomic DNA removed by TURBO DNase (Ambion, Austin, TX), per manufacturers' supplied protocols. cDNA was synthesized using qScript cDNA SuperMix according to the manufacturer's instructions (Quanta BioSciences, Gaithersburg, MD). RT-PCR was performed with Power SYBR Green Master Mix (Invitrogen, Grand Island, NY) on an ABI 7500 Fast Real-Time PCR system using the following cycling parameters: 2 min at 95°C for initial denaturation, 40 cycles consisting of 10 s at 95°C, and 1 min at 60°C for primer annealing and extension. Melt curves were performed to confirm the specificity of primers and the absence of primer dimers. Expression levels were normalized to the housekeeping *rpoD* gene encoding s^70^.

### Multiple sequence alignment and phylogenetic analysis of *ctrA* gene

Sequences of *ctrA* homologues from selected *Alphaproteobacteria* were downloaded from GenBank and aligned using ClustalW2 (http://www.ebi.ac.uk/Tools/msa/clustalw2/). The phylogenetic tree was constructed using software MEGA 4.0 (http://www.megasoftware.net/). BOXSHADE was used to determine the degree of residue shading (www.ch.embnet.org/software/BOX_form.html).

### Statistical analysis

Unpaired Student's *t* test was used to calculate P values.

## Results

### The KLH11 *cckA*, *chpT* and *ctrA* genes are non-essential and control flagellar motility

**Figure 1:**
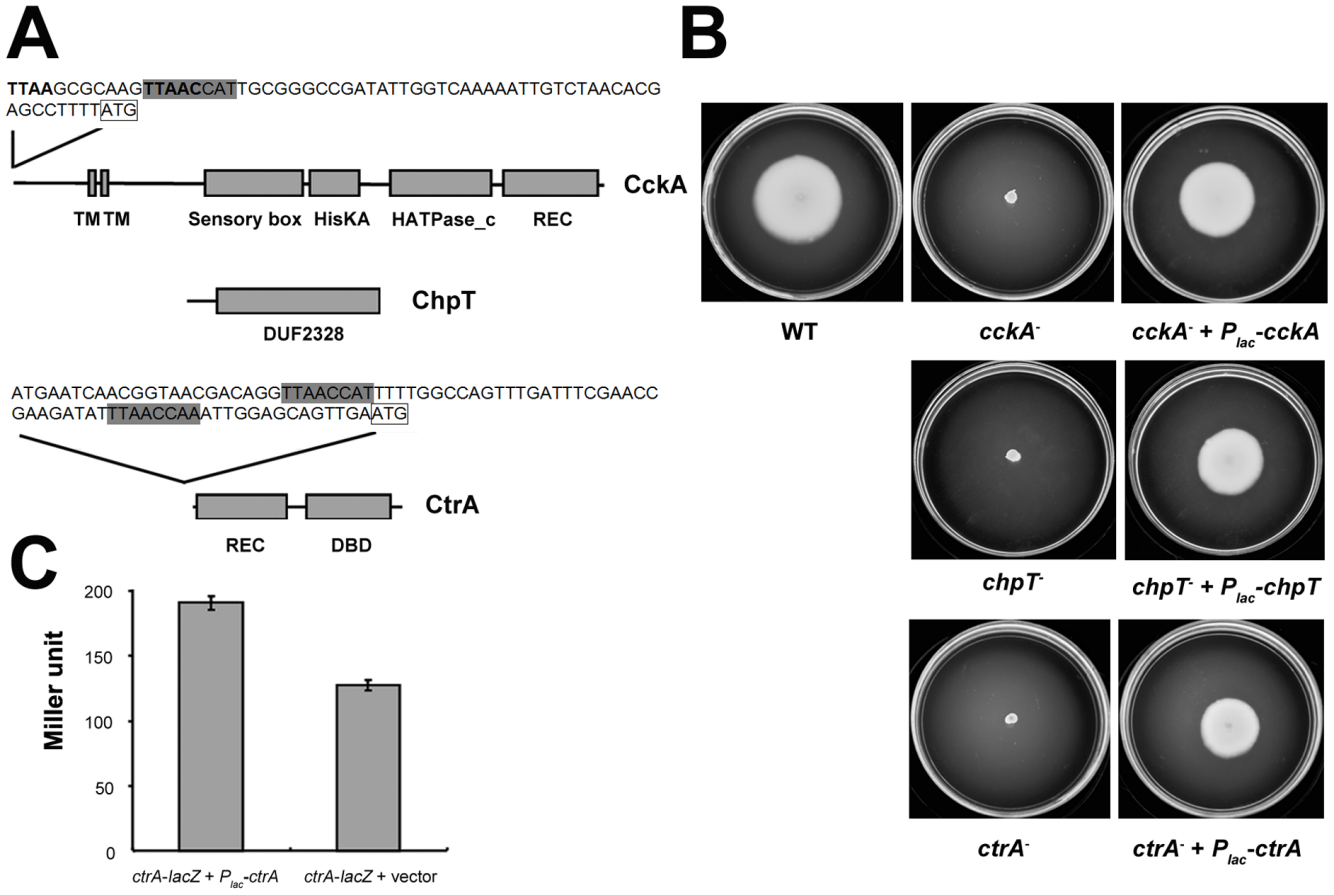
Structure, function and regulation of CckA, ChpT and CtrA. **A) Diagram of the predicted CckA protein, ChpT protein and CtrA protein.** The N-terminus is shown at the left and the C-terminus is shown at the right. TM = transmembrane domain, HisKA = histidine kinase A dimerization/phosphoacceptor domain, HATPase_c = histidine kinase-like ATPase domain, REC = signal receiver domain. DUF2328 is a Pfam domain with unknown function. DBD = DNA binding domain. The length of line was drawn according to scale. The partial promoter regions of the *cckA* and *ctrA* genes are shown on top of the lines. CtrA full recognition site (TTAAN7TTAAC) is in bold and both the CtrA half recognition site (TTAACCAT) and the region that has one mismatch are in grey. The start codon is boxed. **B). Swimming motility assays.** Wild-type KLH11 and derivatives were inoculated on MB2216 (supplemented with 0.25% agar) swim agar plates for 8 days at 28°C. 200 µM IPTG was added to the media. The results are representative of several independent experiments each with three biological replicates. **C).** **CtrA autoregulates its own expression.** *P_lac_-ctrA* plasmid (pJZ008) was conjugated into the *ctrA* ^−^ mutant (JZ06) and the expression of *ctrA*-*lacZ* was monitored by β-galactosidase assay. The pSRKGm was conjugated into the *ctrA* ^−^ mutant as a negative control. Representative results of several independent experiments each with three biological replicates are presented. Values are averages of assays performed in triplicate and error bars are standard deviations.

Annotation of the KLH11 genome revealed that KLH11 has homologues to each of the *cckA*, *chpT* and *ctrA* genes [11]. The putative KLH11 *cckA* gene (GenBank No. ZP_05124558) encodes a 763 amino acid (aa) protein which shares 48% identity at 52% coverage over its C-terminus (367–763) to the *cckA* gene in *C. crescentus*. The N terminus of KLH11 CckA (1–366 aa) has no similarity to that of the *Caulobacter* CckA and had two transmembrane regions predicted by http://www.sbc.su.se/~miklos/DAS/. Domain scans using http://www.ebi.ac.uk/Tools/pfa/iprscan/suggest that the KLH11 CckA protein has a sensory box (273–383 aa), a HisKA domain (394–457 aa), HATPase_c domain (500–620 aa) and REC domain (645–758 aa) (Fig. 1A). The domain organization of KLH11 CcKA is very similar to that of *C. crescentus* CckA [34]. Furthermore, the histidine residue at position 402 and the aspartate residue at position 697 correspond to the conserved phosphorylation sites, histidine 322 and aspartate 623 of *Caulobacter* CckA.

The ChpT Hpt homologue in KLH11 (GenBank No. ZP_05124304) encodes 204 aa and shares 34% identity at 58% coverage (13–131 aa) with *C. crescentus* ChpT, including a histidine at position 24 corresponding to the conserved histidine at position 61 of *Caulobacter* ChpT [35]. It has a hypothetical domain DUF2328 (30–204 aa) conserved in bacteria. The CtrA homologue from KLH11 (GenBank No. ZP_05124475) encodes 238 aa and shares 74% identity across its length with that of *C. crescentus*. It has a receiver domain in the N-terminus (3–112 aa) and a DNA binding domain (145–221 aa) in the C-terminus (Fig. 1A). The phosphorylation site aspartate in KLH11 is also conserved compared to other *ctrA* homologues (Fig. S1).

To test whether *cckA, chpT and ctrA* genes are essential, we attempted to generate Campbell insertions to disrupt the KLH11 *cckA*, *chpT* and *ctrA* genes using the pVIK112 suicide plasmid [27] carrying truncated, internal fragments of each of the three genes (nt 1031–1537 of the *cckA* gene, nt 24–478 of the *chpT* gene, nt 55–518 of the *ctrA* gene). Presumptive kanamycin resistant (Km^R^) recombinants were readily isolated for all *cckA, chpT and ctrA* genes and the integration of the mutagenic plasmids was confirmed by PCR amplification and sequencing. In contrast to their essential role in *C. crescentus*, growth curves of the *cckA* ^−^, *chpT* ^−^ and *ctrA* ^−^ null mutants were similar to that of wild type KLH11 (data not shown). Taken together, these results show conclusively that the *cckA*, *chpT* and *ctrA* genes in *Ruegeria* sp. KLH11 are non-essential and do not affect bacterial growth under laboratory conditions.

We tested the c*ckA* ^−^, *chpT* ^−^ and *ctrA* ^−^ null mutants on Marine Broth 2216 (supplemented with 0.25% agar) swim plates and found that these three null mutants cannot migrate from the inoculation site, unlike the wild-type *Ruegeria* sp. KLH11 that demonstrates motility under these test conditions (Fig. 1B). Provision of plasmid-borne *cckA*, *chpT* and *ctrA* genes expressed from the *lac* promoter (*P_lac_*-*cckA*, pJZ006; *P_lac_-chpT*, pJZ007; *P_lac_-ctrA*, pJZ008) was able to partially restore motility in the corresponding *cckA^−^*, *chpT* ^−^ and *ctrA^−^* null mutants in the presence of 200 µM IPTG to induce *P_lac_*. Microscopic examination of these liquid cultures also revealed no detectable motility for these three mutants (data not shown). Results of a flagellar stain of stationary cultures showed that these three mutants did not synthesize any flagella, in contrast to the wild type (Fig. S2A). Antiserum raised against whole flagella from *C. crescentus*, a related alpha-proteobacterium, was able to recognize KLH11 flagellin protein encoded by the *fliC* gene, of approximately 41.5 kDa in size [11], [19] and was used in the western blot assay. Results showed that none of the three null mutants produced any detectable flagellin protein (Fig. S2B).

### CtrA regulates motility-related gene expression but not cell cycle-related genes

CtrA regulates expression of a wide range of genes involved in different cellular processes in several bacterial species [9], [36], [37]. We used quantitative reverse transcription-PCR (qRT-PCR) to detect the expression differences of motility-related genes between wild type KLH11 and *ctrA* ^−^ mutant. Five genes: *motB, fliL, flgB, flgJ* and *fliG*, which are the first genes in their predicted motility-related operons, and the *flhA* gene, which is the second gene in its operon (the presumptive first gene is not homologous to any known motility genes), were chosen for analysis. The flagellin gene (*fliC*) was also selected for testing. All the predicted motility-related genes we tested were significantly decreased in the *ctrA^−^* mutant, ranging from 9- to 93- fold differences between wild type KLH11 and the *ctrA* ^−^ mutant (Table 1). Provision of plasmid-borne KLH11 CtrA (*P_lac_-ctrA*) into the *ctrA^−^* mutant restored the expression levels almost to those in wild type KLH11. We similarly tested CtrA regulation of the cell cycle related genes *ftsZ* (GenBank No. ZP_05121748) and *ccrM* (GenBank No. ZP_05124520), orthologues of which are CtrA-controlled in *C. crescentus*. It is clear that under the conditions we examined, CtrA does not regulate the expression of the *ftsZ* or *ccrM* genes (Table S3).

**Table 1:** Quantification of motility-related gene expression by qRT-PCR.

Gene Name	Putative Gene Class	Wild-type^a^^,^ b	*ctrA* ^−a^	Plasmid-borne *ctrA* a ^,^ b	fold change WT/*ctrA* ^−^
*fliL*	2	456 (91)	12 (3)	535 (255)	38
*fliF*	2	323 (65)	5 (1)	299 (75)	65
*flgB*	2	746 (219)	8 (3)	602 (176)	93
*flhA*	2	9 (3)	1 (1)	7 (1)	9
*flgJ*	3	154 (28)	10 (1)	174 (67)	15
*fliC*	3	93 (28)	7 (<1)	51 (16)	13
*motB*	3	285 (56)	16 (2)	323 (103)	18

a Value relative to the *rpoD* gene. Average of three biological replicates (standard deviation). The values are multiplied by 1000.

b All P values are <0.05 when compared the indicated column to the *ctrA* ^−^ column.

### CtrA autoregulates its own transcription but not that of the *cckA* gene

KLH11 CtrA has an identical amino acid sequence in the putative helix-turn-helix DNA sequence recognition region to that of *C. crescentus* (Fig. S1). The DNA sequences with which this CtrA protein interacts, have been well characterized as TTAA-N7-TTAAC (full site) and TTAACCAT (half-site) in *C. crescentus*. However, it is clear that the CtrA protein can also bind to more degenerate sequences that appear to share only the TTAA sequences [9]. Examination of the sequences upstream of the predicted *ctrA* translation start site revealed a putative half site (62 bp upstream of the predicted translational start, Fig. 1A) and thus we tested whether CtrA autoregulates its own expression. The plasmid integration used to disrupt the *ctrA* gene (pJZ005 derived from pVIK112) simultaneously generates a transcriptional fusion to the disrupted gene [27]. The *P_lac_-ctrA* plasmid (pJZ008) and a vector control (pSRKGm) were conjugated in parallel into strain JZ06 (*ctrA*-*lacZ*). Under the 200 µM IPTG induction of the *P_lac_-ctrA* plasmid, there was a statistically significant, yet modest ∼50% increase of *ctrA* expression (P<0.05) compared to the vector control (Fig. 1C).

Inspection of the *cckA* upstream sequences for CtrA binding sites identified one putative CtrA full recognition site (62 bp upstream of the predicted translational start) and one half site (51 bp upstream of the predicted translational start), although these two sites overlap (Fig. 1A). We used a similar approach to that described above to test whether CtrA affects *cckA* expression. However, we reasoned that the *cckA* gene might be required to generate the phosphorylated CtrA capable of regulating the *cckA* promoter. Therefore instead of using the strain JZ04 with a disrupted *cckA* gene fused to *lacZ* on the integrated plasmid, we created strain JZ13 in which the wild type *cckA* gene is retained, but transcriptionally fused to *lacZ* carried on the integrated plasmid (see Experimental procedures). Introduction of the *P_lac_-ctrA* plasmid into JZ13 did not alter *cckA* expression (Table S4). Inspection of the *chpT* upstream region for the CtrA binding sites did not identify sequences similar to either the full site or the half site.

### Cross complementation between KLH11 and *Agrobacterium tumefaciens* homologues

**Figure 2:**
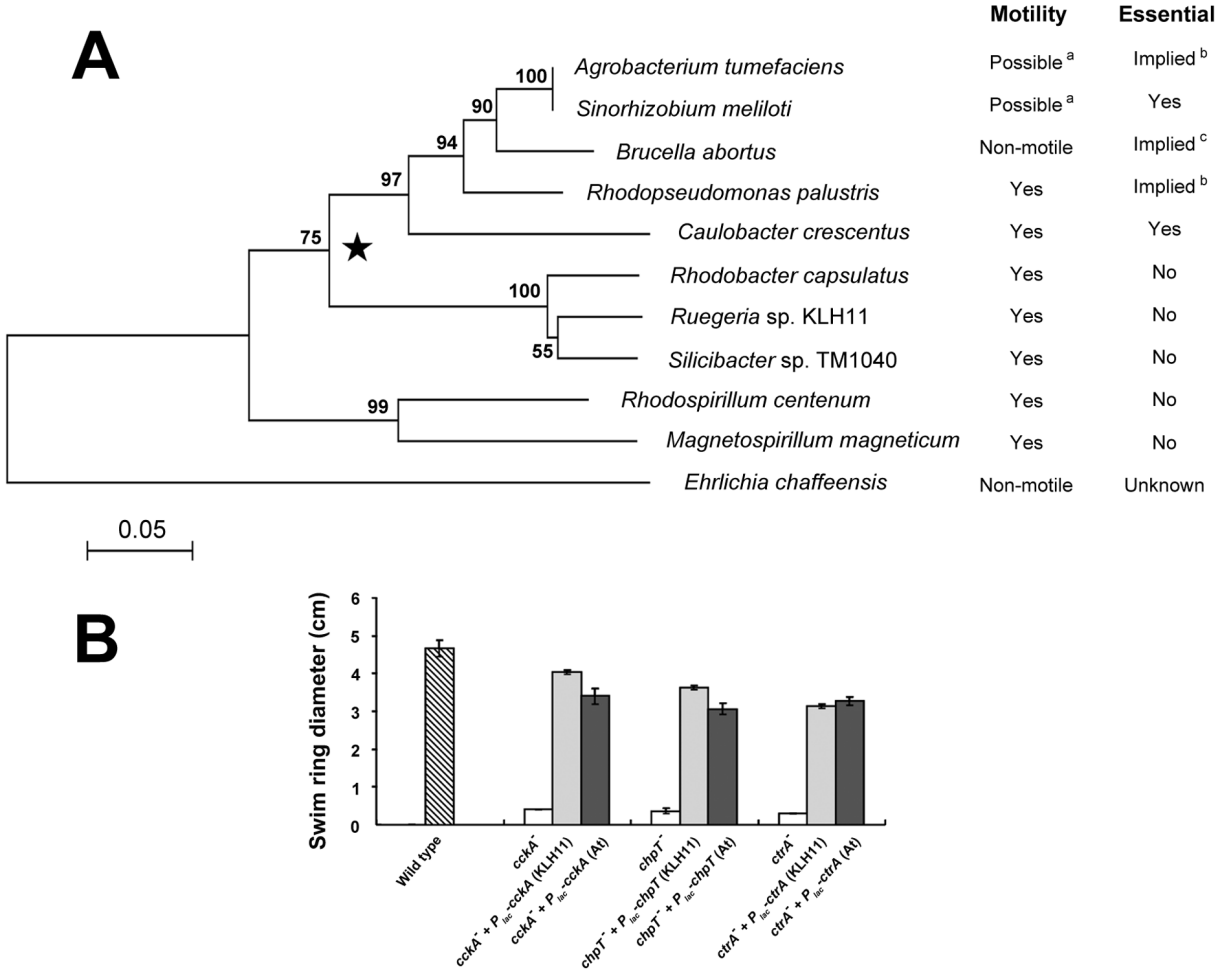
Phylogeny of the CtrA protein cross-complementation between KLH11 and *A. tumefaciens*. **A) Phylogenetic analyses of CtrA from members of alpha-** ***Proteobacteria*** **.** CtrA sequences from bacterial species in which CtrA has been studied were chosen for phylogenetic anaylsis. The sequences used were *A. tumefaciens* C58 (GenBank Accession No. NP_355385), *B. abortus* (AAL86376), *C. crescentus* (NP_421829), *E. chaffeensis* (YP_507798), *Magnetospirillum magneticum* AMB-1 (YP_419992), *Rhodopseudomonas palustris* (NP_946978), *Rhodobacter capsulatus* (AAF13177), *Rhodospirillum centenum* (YP_002297962), *Ruegeria* sp. KLH11 (ZP_05124475), *Silicibacter* sp. TM1040 (YP_613394) and *Sinorhizobium meliloti* (NP_386824). The star indicates the divergence between organisms in which CtrA is essential or implied to be essential and in which CtrA does affect viability, which was originally proposed by Green et al. [37] a = motility-related genes are enriched in putative CtrA binding sites ([12]; b = unable to obtain a ctrA deletion mutant without providing an extra copy of the *ctrA* gene (J.E. Heindl and C. Fuqua, unpublished) [36]; c = gene target (*ccrM*) of CtrA is essential [50]. The scale bar indicates the number of amino acid substitutions per site. **B) Cross complementation of motility between KLH11 and** ***A. tumefaciens*** **homologues.** Wild-type KLH11 (EC1) and derivatives were inoculated on MB2216 (supplemented with 0.25% agar) swim agar plates for about 8 days at 28°C. 200 µM IPTG was added to the media. The diameter of the swim ring was measured. Parentheses indicate from which species the relevant homologue is used (At stands for *A. tumefaciens*). Values are averages of assays performed in triplicate and error bars are standard deviations.

Phylogenetic analysis showed that the *ctrA* gene of KLH11 falls into the non-essential group (Fig. 2A) of the two proposed by Greene *et al.* (2012). In contrast, the *ctrA* homologue in *A. tumefaciens* is within the predicted essential group, and disruption of this gene is not possible unless a second copy of *ctrA* is also provided (J.E. Heindl and C. Fuqua, unpublished). We introduced an expression plasmid that carries the full-length *ctrA* gene from *A. tumefaciens* expressed from the *P_lac_* promoter (pJEH028) into the *Ruegeria* sp. KLH11 *ctrA* ^−^ mutant (JZ06) and determined if this *A. tumefaciens* CtrA protein can restore motility. Strikingly, provision of the *A. tumefaciens* CtrA can restore motility in the *ctrA^−^* mutant to the same extent as the KLH11 *ctrA* gene (P>0.05) (Fig. 2B). Similarly, the plasmids that carry the full-length *cckA* gene (pJEH010) and *chpT* gene (pJEH027) of *A. tumefaciens* were introduced into the KLH11 *cckA^−^* (JZ04) and *chpT* ^−^ (JZ05) mutants, respectively, and also partially restored motility at levels slightly lower than the KLH11 *cckA* and *chpT* genes can (P<0.05) (Fig. 2B). However, the *A. tumefaciens cckA* plasmid failed to restore motility in the the KLH11 *cckA^−^* mutant in ca. 30% of our experiments suggesting there may be additional variables that we were not controlling (data not shown).

### The SsaRI quorum sensing system regulates the transcription of *ctrA*, *chpT* and *cckA* genes

**Figure 3:**
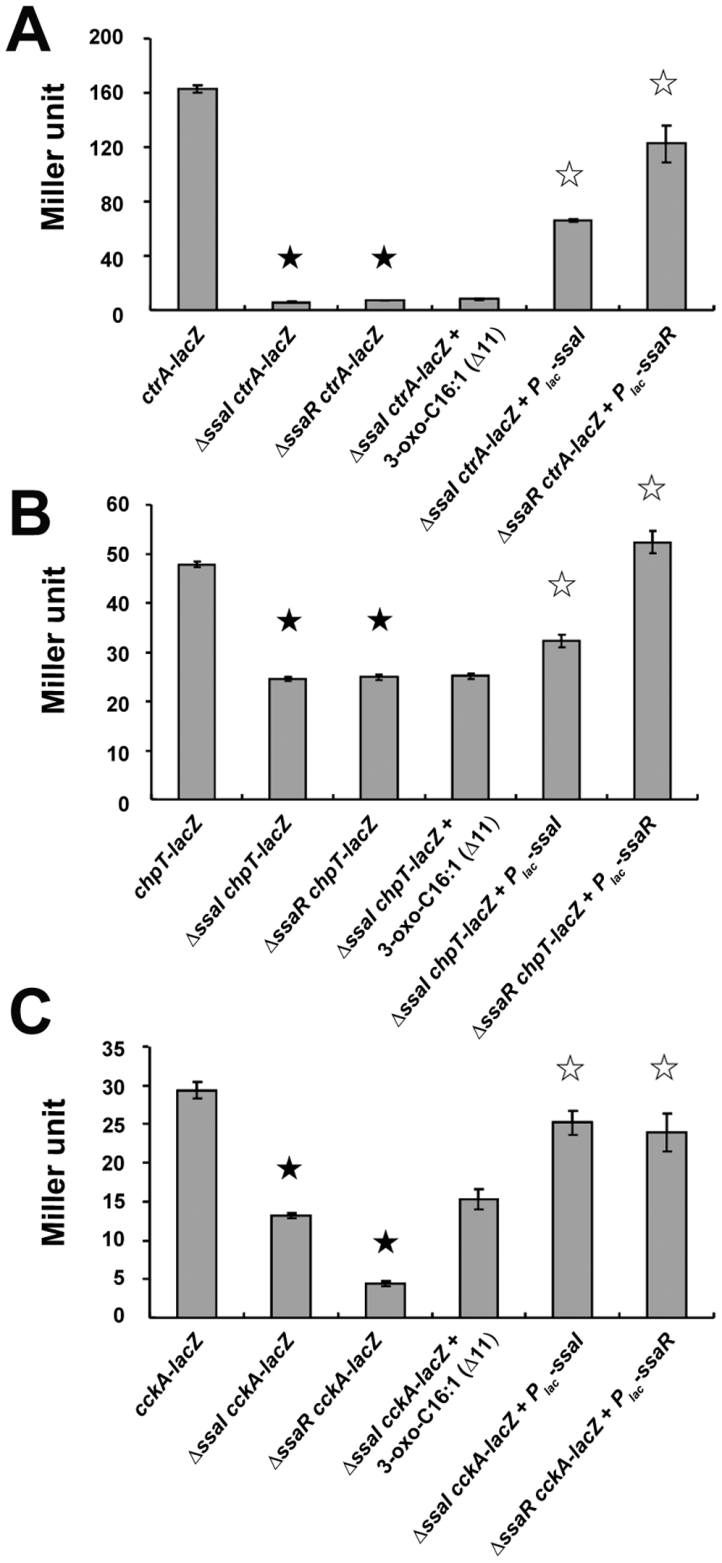
Regulation of *cckA, chpT* and *ctrA* gene expression by the *ssaRI* **system.** Results of β-galactosidase assays in detecting the expression *of ctrA–lacZ* (A), *chpT-lacZ* (B) and *cckA-lacZ* (C) in Δ*ssaI* and Δ*ssaR* mutants. Plasmids *P_lac_-ssaI* (pEC108) and *P_lac_-ssaR* (pEC112) were conjugated into the Δ*ssaI* and Δ*ssaR* mutants, respectively, to restore the expression of the *ctrA*, *chpT* and *cckA* genes. 2 µM 3-oxo-C16:1 Δ11-HSL was added into Δ*ssaI ctrA-lacZ*, Δ*ssaI chpT-lacZ and* Δ*ssaI cckA-lacZ* strains, respectively. Filled asterisks indicated statistically significant differences between the indicated strain and wild-type quorum sensing strain. Unfilled asterisks indicated statistically significant differences between the quorum sensing complemented strains and quorum sensing mutants for the expression of the *ctrA*, *chpT* and *cckA* genes. Representative results of several independent experiments each with three biological replicates are presented. Values are averages of assays performed in triplicate and error bars are standard deviations.

In KLH11, the QS circuit *ssaRI* controls flagellar motility [19]. We therefore tested whether *ssaRI* regulates the expression of the *ctrA*, *chpT* and *cckA* genes. Campbell-type insertions in the *ctrA*, *chpT* and *cckA* genes using the suicide vector pVIK112 with internal fragments of each gene created null mutants and simultaneously generated *lacZ* transcriptional fusions to the disrupted gene [27]. We used β-galactosidase assays to compare the expression of *ctrA*, *chpT* and *cckA* genes in Δ*ssaI* and Δ*ssaR* deletion mutants, respectively, from cultures grown to an OD_600_∼0.6. Expression of the *ctrA-lacZ* fusion was decreased approximately 25-fold in both the *ΔssaI* and *ΔssaR* mutants (Fig. 3A; P<0.01). The *chpT-lacZ* (Fig. 3B) and *cckA-lacZ* (Fig. 3C) fusions were also decreased significantly for the *ΔssaI* and *ΔssaR* mutants, but less dramatically for *chpT* (2 fold for both mutants; both with P<0.05) and 2–6 fold for *cckA* (*ΔssaI,* 2-fold, P<0.05; *ΔssaR*, 6-fold, P<0.05). Ectopic expression of plasmid-borne *P_lac_-ssaI* and *P_lac_*-*ssaR* restored the expression of *cckA, chpT and ctrA* genes in the corresponding *ssaI* and *ssaR* mutants to levels closer to wild type.

AHL synthase gene mutants can usually be rescued by exogenous addition of the appropriate AHL. The KLH11 *ssaI* mutant motility defect can be partially restored with exogenous addition of synthetic 3-oxo-C16:1 Δ11-HSL, an AHL similar to that specified by SsaI [19]. We therefore tested whether this AHL could rescue *ctrA*, c*hpT* and *cckA* expression in the corresponding mutants. Surprisingly, addition of this AHL failed to restore the expression of the *ctrA*, *chpT* or *cckA lacZ* fusions in the *ΔssaI* mutant (P>0.05, Fig. 3A–C). In all of these Campbell insertions, generation of the fusion also disrupts the gene. To examine QS-dependent expression of these genes in an otherwise wild type background, we introduced the following plasmid-borne fusions into the *ΔssaI* strain: *P_ctrA_-lacZ* (pJZ011), *P_chpT_-lacZ* (pJZ010) and *P_cckA_-lacZ* (pJZ009). The expression of these *lacZ* fusions was monitored by β-galactosidase assays in the presence and absence of 2 µM 3-oxo-C16:1 Δ11-HSL. A 2.5-fold increase for *P_ctrA_-lacZ* (*P*<0.05) and 4-fold increase for *P_cckA_-lacZ* (P<0.05) was observed in the presence of 2 µM 3-oxo-C16:1 Δ11-HSL (Table 2) while we did not observe a significant increase for *P_chpT_-lacZ* fusion (data not shown).

**Table 2:** Exogenous AHLs complement for the lack of a functional *ssaI* gene in the indirect activation of the *cckA* and *ctrA* genes.

	Wild type^a^		Δ*ssaI* a	
	No AHL	+AHL^b^	No AHL	+AHL^b^
*P_cckA_-lacZ* (pJZ009)	48 (2.3)	48.5 (2.6)	1.6 (0.2)	8 (0.3)^c^
*P_ctrA_-lacZ* (pJZ011)	182.6(15.7)	182.6 (15.6)	2.9 (0.6)	7.1 (0.4)^c^

a β-Galactosidase activity was expressed in Miller unit. Average of three biological replicates (standard deviation).

b 3-oxo-C16:1 Δ11-HSL (2 µM) was added.

c P<0.05 when the expression level with AHL is compared to that without AHL in the Δ*ssaI* strain.

### SsaRI regulate *ctrA*, *chpT* and *cckA* expression indirectly

The gene expression experiments in KLH11 did not allow us to distinguish direct or indirect QS regulation of the CckA-ChpT-CtrA pathway. We therefore electroporated plasmids carrying *P_ctrA_-lacZ* (pJZ011) and *P_lac_-ssaR* (pEC112) into the AHL^−^ *A. tumefaciens* NTL4 (Ti-plasmidless) derivative to test whether the QS-dependent expression of *ctrA* was due to SsaR-dependent activation of the *ctrA* promoter. In this same background, SsaR and 3-oxo-C16:1 Δ11-HSL strongly activate the expression of the *P_ssaI_* promoter [19]. *A. tumefaciens* NTL4 harboring *P_ctrA_-lacZ* (pJZ011) plus a vector (pBBR1-MCS5) was used as a negative control. Expression of the *P_ctrA_-lacZ* fusion was unaffected by addition of 2 µM 3-oxo-C16:1 Δ11-HSL (Table S5). These results indicate that SsaR indirectly regulates the expression of *P_ctrA_-lacZ* and that an intermediary regulator(s) must exist. We used the same approach to test the regulation of *chpT* (*P_chpT_-lacZ*, pJZ010) and *cckA* (*P_cckA_-lacZ*, pJZ009) by SsaR with 2 µM 3-oxo-C16:1 Δ11-HSL. These findings suggest that SsaR and 3-oxo-C16:1 Δ11-HSL do not directly activate the expression of *ctrA, chpT* and *cckA* genes (Table S5).

### Ectopic expression of ctrA restores motility to the QS deletion mutant

**Figure 4:**
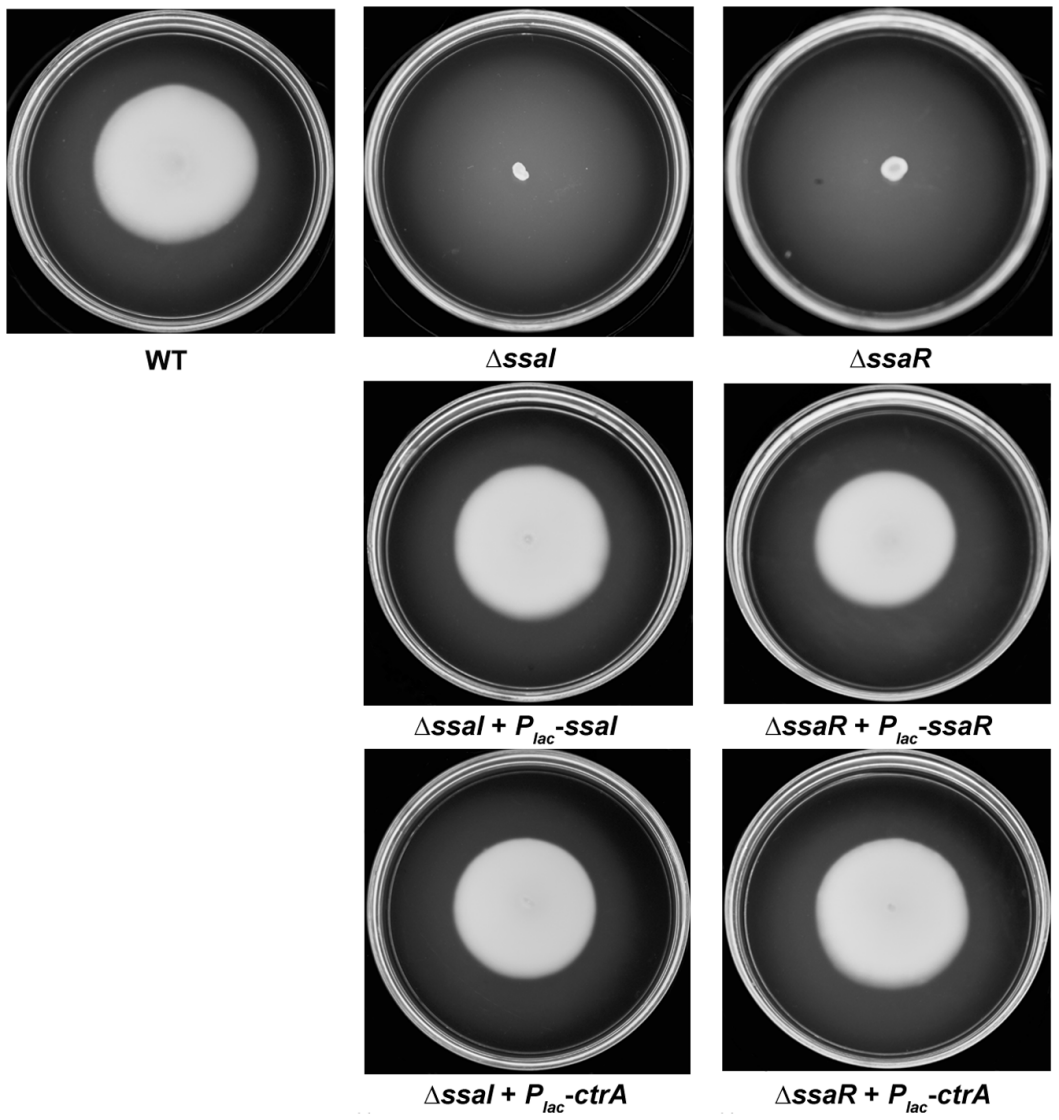
Suppression of motility defects in Δ*ssaI* and Δ*ssaR* mutants by CtrA. *P_lac_-ctrA* plasmid (pJZ008) was conjugated into Δ*ssaI* and Δ*ssaR* mutants, respectively. The conjugants were selected and inoculated on swim agar plates for 8 days at 28°C. 200 µM IPTG was added to the media. The Δ*ssaI* mutant complemented with *P_lac-_ssaI* (pEC108) and the Δ*ssaR* mutant with *P_lac-_ssaR* (pEC112) were used as positive controls. Wild type KLH11 (EC1) was used as a positive and the Δ*ssaI* and Δ*ssaR* strains were used as negative control. The results were representatives of several independent experiments each with three biological replicates.

Provision of neither the plasmid-borne *P_lac_-ssaI* (pEC108) nor *P_lac_*-*ssaR* (pEC112) to the corresponding mutants (Δ*ssaI cckA* ^−^, Δ*ssaI chpT*,^−^ Δ*ssaI ctrA* ^−^ and Δ*ssaR cckA^−^*, Δ*ssaR chpT* ^−^, Δ*ssaR ctrA* ^−^) restored motility (Fig. S3), although they did restore nearly wild type expression levels for each *lacZ* fusion (Fig. 3). This is due to the disruption of the targeted gene by the Campbell insertions. Consistent with our previous studies [19] however the *P_lac_-ssaI* (pEC108) or *P_lac_*-*ssaR* (pEC112) plasmids effectively complement motility in the Δs*saI* and Δ*ssaR* mutants, respectively (Fig. 4). This suggests that *cckA*, *chpT* and *ctrA* are required for motility and act downstream of the *ssaRI* system. Accordingly, IPTG-induced expression of the *P_lac_-ctrA* (pJZ008) in Δs*saI* and Δ*ssaR* did however restore motility (Fig. 4). Controls with the vector alone did not correct the motility defect in any of these derivatives (data not shown). Furthermore, the *P_lac_-ctrA* plasmid could not restore motility in the *ΔssaI cckA* ^−^, Δ*ssaI chpT* ^−^, Δ*ssaR cckA* ^−^ or Δ*ssaR chpT* ^−^ mutants, respectively (Fig. S4). Thus, *cckA* and *chpT* genes are required for the suppression of the *ΔssaI* or *ΔssaR* mutant motility defects by CtrA. Furthermore, *P_lac_-ctrA* (pJZ008) was unable to restore motility to the Δ*ssaI* or Δ*ssaR* that were also disrupted for the flagellin gene *fliC* (data not shown), confirming that CtrA imparts its influence on motility through the flagellar system.

## Discussion

The *cckA-chpT-ctrA* phosphorelay system has been well characterized in *C. crescentus* in which the expression levels of 144 genes are affected due to the loss of the *ctrA* gene [10]. However, relatively little is known about this phosphorelay system in the highly abundant marine *Roseobacter* clade. In several alphaproteobacterial systems, *cckA* and *ctrA* are essential, although little is known about the essentiality of the *chpT* gene other than in *C. crescentus, Rhodobacter capsulatus*, and *Silicibacter* sp. TM1040 [13], [35], [38]. However, our results clearly reveal that all the *cckA* ^−^, *chpT* ^−^ and *ctrA* ^−^ mutants have growth rates nearly identical to that of wild type KLH11, demonstrating that they are non-essential under laboratory conditions. This observation is consistent with the function of *cckA* and *ctrA* in *Rhodobacter capsulatus* [39], *Silicibacter* sp. TM1040 [13], and *Rhodospirillum centenum* [40]. The *ctrA* gene is not essential in *Magnetospirillum magneticum* AMB-1 [37] but the essentiality of *cckA* has not been examined. Moreover, qRT-PCR revealed that CtrA did not regulate the expression of the *ftsZ* or *ccrM* genes in KLH11 (Table S3). In *C. crescentus* CtrA directly regulates the expression of genes involved in cell division such as *ftsZ*, encoding the primary division protein, and *ccrM*, a DNA methyltransferase that modifies sequences at the replication origin to coordinate the timing of genome replication with the cell division cycle [9]. Taken together, the non-essentiality of *ctrA* and the lack of regulation for *ftsZ* and *ccrM* indicate that CtrA probably does not play a role in cell cycle control in KLH11.

Our findings provide evidence that CckA, ChpT and CtrA activate swimming motility and control the biosynthesis of flagella (Figs 1 and S2), similar to a portion of their roles in *C. crescentus*, although the intimate relationship between the cell cycle and flagellation confounds this role [8], [10]. Furthermore, qRT-PCR revealed *ctrA* to regulate all the motility-related genes that were checked. We searched for both full and half *ctrA* binding sites using the motifs as determined from Laub et al. [9] in the upstream regions of these motility-related genes, but no sequences with high similarity were identified. This is similar to the situation in *Rhodobacter capsulatus*, in which expression of motility related genes is decreased in a Δ*ctrA* strain but none of these genes have clear *ctrA* binding motifs [36].

Identification of presumptive CtrA recognition sites in the upstream of *ctrA* suggested feedback on its own transcription. Our results indeed showed the positive feedback on the *ctrA* expression. This is consistent with findings in *C. crescentus*, although in that bacterium CtrA negatively regulates one promoter (P1) and positively regulates a second (P2) [41]. It is unclear whether or not the KLH11 *ctrA* gene has multiple promoters. In *S. meliloti*, CtrA∼P can bind proximal to its two *ctrA* promoters, presumably regulating the transcription of the *ctrA* gene [42]. Similarly, in the obligatory intracellular pathogen *Ehrlichia chaffeensis*, the CtrA protein can also bind proximal to its own promoter [43]. In contrast, for *Rhodobacter capsulatus*, *ctrA*-binding site was identified in the *ctrA* promoter regions [39], but CtrA does not affect its own transcription [44]. A search of the promoter region of the *cckA* gene also identified presumptive *ctrA* binding sites (Fig. 1A), suggesting that CtrA could potentially regulate the expression of the *cckA* gene. However, provision of *ctrA* in the KLH11 derivative with the integrated *cckA-lacZ* that maintains an intact copy of *cckA* (JZ13) does not affect *cckA* gene expression (Table S4). This is similar to *Rhodobacter capsulatus*, in which the transcription of *cckA* gene is not affected in a Δ*ctrA* strain, although interestingly, loss of the *ctrA* gene leads to a decrease of CckA protein levels [36]. We do not know whether the loss of the *ctrA* gene would affect the amount of CckA protein in KLH11. This also emphasizes that the presence of upstream sequences with similarity to CtrA binding sites does not necessarily mean that the associated gene (in this example *cckA*) is regulated by CtrA. Taken together, this likely reflects the limits on current understanding of what comprises a CtrA binding site outside of *C. crescentus*.

Greene et al. [37] proposed two groups of CtrA in the *Alphaproteobacteria*: in one group *ctrA* is essential and in the other it is non-essential, but in both groups it exerts control over motility. KLH11 *ctrA* clearly falls into the non-essential group by sequence comparisons (Fig. 2A). Interestingly, although the *cckA*-*chpT*-*ctrA* pathway is essential in *A. tumefaciens* [29], plasmid-borne expression of each of these *A. tumefaciens* genes can cross-complement the corresponding mutants in KLH11 for their impact on motility. This cross-complementation suggests that the functionality of this pathway is well conserved and its role in controlling motility is ancestral among the *Alphaproteobacteria*. These proteins have retained their basic activities, even though the influence of this pathway can be expanded to include essential functions. It remains unclear whether the pathway's essentiality is derived or ancestral among the *Alphaproteobacteria*. The CckA protein from *A. tumefaciens* shows inconsistent complementation in the KLH11 *cckA* ^−^ mutant, which hints at an additional signal(s) that may impact the activity of the *A. tumefaciens* CckA protein.

Our results clearly show that the *cckA-chpT-ctrA* phosphorelay system is indirectly transcriptionally regulated by the SsaRI quorum sensing circuit. In *C. crescentus*, the transcription of the *cckA* gene is cell cycle dependent, but not affected by CtrA [9], [10]. Moreover, the level of the CckA protein is constant during the cell cycle whereas the phosphorylation of CckA is subject to temporal and spatial regulation [34], [45]. We do not know what signal(s) it is to which the CckA protein responds. However, it is plausible that the CckA protein may sense population density-associated signals and thus it can coordinate the activation of motility with the cell density. Of note, the Δ*ssaR* mutant exhibits a more profound deficiency in the *cckA* expression than the Δ*ssaI* mutant (Fig. 3C). One explanation is that SsaR is able to respond to the AHL levels synthesized in the *ΔssaI* mutant, in which the *ssbRI* system remains genetically intact [19]. Meanwhile, the activity of CtrA, a key factor in driving the cell cycle, is tightly regulated at the levels of transcription, phosphorylation, degradation, and protein-protein interaction [46]. On the transcriptional level, the *C. crescentus ctrA* gene is activated by cell-cycle master regulator GcrA [47]. In *Rhodobacter capsulatus*, it was found that the LuxR-holomogue GtaR indirectly represses the transcription of *ctrA* while the AHL synthesized by GtaI derepresses its transcription [44], [48]. However, it was unclear whether QS affects the transcription of *cckA* and *chpT* genes in this bacterium.

Interestingly, we can complement the expression of *ctrA*, *chpT* and *cckA*–*lacZ* fusions as Campbell insertions in the Δ*ssaI* background by providing the *ssaI* gene in trans but were not able to restore their expression by addition of exogenous synthetic 3-oxo-C16:1 Δ11-HSL (Fig. 3A–C). It is known that addition of AHL into Δ*ssaI* is able to partially restore motility [19] and data in this study clearly show that CtrA acts downstream of the *ssaRI* system to control flagellar assembly and motility. We reason that two factors can contribute to this observation: 1) The long chain AHL we added might not be able to partition into the cell from exterior efficiently due to its hydrophobicity. It has been shown that the long chain AHLs preferentially associate with the cell rather than being released extracellularly and that AHLs that partition into the cell membrane may not function as signals [49]. Addition of the same AHL can stimulate the expression of the *ssaI* gene [19]; however, the stimulatory effect of AHL on *ssaI* might not be propagated onto the *cckA*-*chpT*-*ctrA* effectively because of the indirect regulatory link between *ssaRI* and *cckA*-*chpT*-*ctrA* 2) There might be some positive feedback on the *ssaRI* system by this pathway. In the Campbell insertion, the gene is disrupted. It is possible that an intact copy of this pathway is required for optimal expression. Indeed, the significant, yet weak activation of the plasmid borne fusions by the addition of AHL in the Δ*ssaI* strain in which *cckA*-*chpT*-*ctrA* are intact supports this speculation (Table 2).

**Figure 5:**
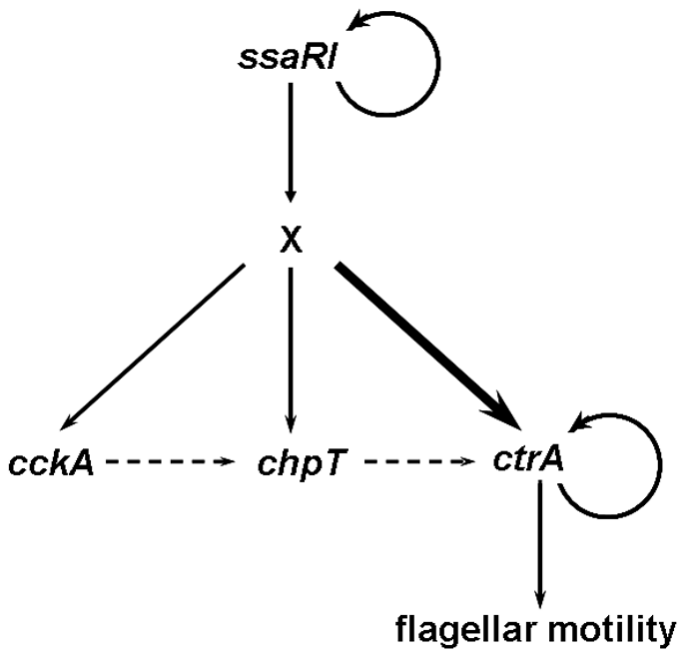
A tentative model for the *ssaRI* to *cckA-chpT-ctrA* regulatory circuit to control KLH11 flagellar motility. The solid lines with the arrows indicate activation. The dashed line with arrows indicates the potential phosphate flow from CckA to CtrA via ChpT. The curved lines with arrows around CtrA and SsaRI indicate positive feedback loops. The “X” indicates the unknown regulator(s). The thicker line from X to *ctrA* indicates stronger regulatory effect.

Our data supported that the phosphorylation of CtrA is required for motility control, which is consistent with previous studies [10], [38], [40], because ectopic expression of *ctrA* in either the Δ*ssaI* or Δ*ssaR* mutants can restore motility while provision of CtrA into each of these four mutants: Δ*ssaI cckA* ^−^, Δ*ssaR cckA* ^−^, Δ*ssaI chpT* ^−^, and Δ*ssaR chpT* ^−^ mutants does not restore motility. Taken together, our data support the model shown in Fig. 5. SsaRI acts upstream of the *cckA*-*chpT*-*ctrA* phosphorelay system and indirectly regulates the transcription of all the three genes, most dramatically through *ctrA* expression. CckA and ChpT are required via presumptive phosphotransfer to CtrA, which positively feeds back on its own expression, and controls flagellar assembly and motility. CtrA could thus be the potential flagellar master regulator in KLH11. This is similar to the model reported in the rice pathogen *B. glumae*, in which the *tofRI* QS pathway controls the regulator *qsmR* which in turn directly controls the flagellar master regulator *flhDC* [20]. However, the presumptive regulator that links SsaRI to *ctrA* remains to be identified in KLH11. Our data also suggests that there may be feedback from the CckA-ChpT-CtrA pathway on the SsaRI system.

Roseobacters are a key marine bacterial group with biogeochemical relevance and are also commonly found as symbionts of marine invertebrates, including sponges. Roseobacters are often highly abundant in phytoplankton blooms or near macroalgae and associated with organic particles [2]. Motility is likely to be critical in many of these interactions. Our previous work contributed to understanding the role of quorum sensing in activating motility specifically at high cell density in the sponge-associated Roseobacter, *Ruegeria* sp. KLH11 [19], raising the possibility that this is a widespread mechanism in Roseobacters. In KLH11, flagellar motility is controlled by the SsaRI system and AHL quorum sensing. Surprisingly, the KLH11 genome and that of its relative *R. pomeroyi* DSS-3 lack any recognizable chemotaxis genes [11], [21]. Therefore QS regulation of motility is not simply augmenting the process, but appears to be its primary control mechanism. SsaR and long chain AHLs are required for *cckA*, *chpT* and *ctrA* gene expression, revealing at least a portion of this central control pathway, although additional environmental signals may also function through the CckA-ChpT-CtrA cascade. The work reported here provides a discrete regulatory link between flagellar locomotion and population density in KLH11.

## Supporting Information

Figure S1 **Alignment of KLH11 CtrA amino acid sequence to selected CtrA homologues.** The degree of shading is determined by using software BOXSHADE. The helix–turn–helix DNA-binding motif is boxed with a dashed line. The conserved Asparate residue is indicated with an asterisk above. Amino acid numbers for each CtrA protein are shown on the left. The GenBank accession numbers for sequences used in this alignment are shown in Fig. 2A.
(TIFF)

Figure S2 **Detection of flagella and flagellin in KLH11 and mutants.** **A) Flagellar stain of wild type KLH11,** ***cckA*** **^−^,** ***chpT^−^*** **and** ***ctrA^−^*** **null mutants.** Stained cells from late stage cultures were viewed under phase contrast microscopy with 100X lens. Wild type (EC1), *cckA* ^−^ (JZ04), *chpT* ^−^ (JZ05), *ctrA^−^* (JZ06), Red arrows indicate stained flagella. The bar represents 10 µm. **B) Detection of flagellin in wild type KLH11,** ***cckA*** **^−^,** ***chpT^−^*** **and** ***ctrA^−^*** **null mutants.** Antibody raised against *C. crescentus* whole flagella was used to probe for flagellin. Samples were collected at stationary phase. Flagellin was extracted from 3 ml late stage culture from each of the 4 strains with similar OD_600_. The extraction was dissolved in 100 µl 1X sample buffer and boiled for 5 min. 30 µl was loaded onto each lane. Estimated size of KLH11 flagellin is 43 kDa.
(TIFF)

Figure S3 **The** ***cckA-chpT-ctrA*** **phosphorely system is required for the** ***ssaRI*** **system to control motility.** *P_lac_-ssaI* (pEC108) was conjugated into Δ*ssaI cckA^−^*, Δ*ssaI cphT^−^ and* Δ*ssaI ctrA ^−^* double mutants and *P_lac_-ssaR* (pEC112) was conjugated into Δ*ssaR cckA^−^*, Δ*ssaR cphT^−^ and* Δ*ssaR ctrA ^−^* double mutants, respectively. Strains were inoculated on MB2216 (supplemented with 0.25% agar) swim agar plates for about 8 days at 28°C. The results were representatives of several independent experiments each with three biological replicates.
(TIFF)

Figure S4 **The** ***cckA*** **and** ***chpT*** **genes are required for the function of CtrA.** *P_lac_-ctrA* was conjugated into Δ*ssaI cckA^−^*, Δ*ssaI chpT^−^* ^,^ Δ*ssaR cckA^−^*, and Δ*ssaR chpT^−^* ^,^ respectively. The conjugants were selected and inoculated for swim motility assay as described above. The results were representatives of several independent experiments each with three biological replicates.
(TIFF)

Table S1 **Strains and plasmids used in this study.**
(DOCX)

Table S2 **Primers used in this study.**
(DOCX)

Table S3 **Quantification of** ***ftsZ*** **and** ***ccrM*** **expression by qRT-PCR.**
(DOCX)

Table S4 **Regulation of** ***cckA*** **by** ***ctrA*** **.**
(DOCX)

Table S5 **Expression of KLH11** ***P_cckA_, P_chpT_*** **and** ***P_ctrA_*** **promoters in an AHL^−^ host.**
(DOCX)
