# Supplementary figures and images for: The CckA-ChpT-CtrA Phosphorelay System Is Regulated by Quorum Sensing and Controls Flagellar Motility in the Marine Sponge Symbiont Ruegeria sp. KLH11
Source: PLoS One. 2013 Jun 25;8(6):e66346. doi: 10.1371/journal.pone.0066346 (PMC3692519; doi:10.1371/journal.pone.0066346)

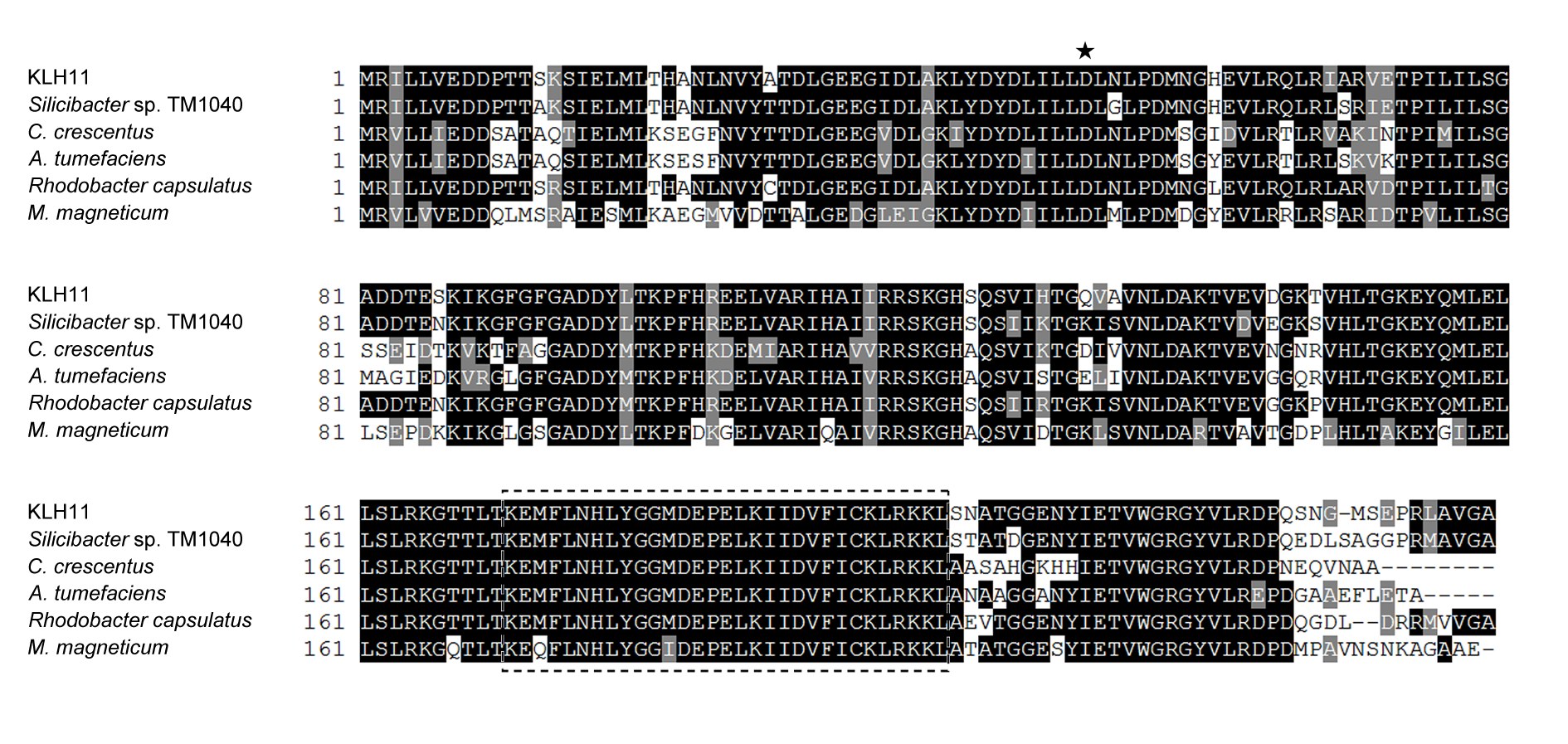

Supplement: Figure S1 — Alignment of KLH11 CtrA amino acid sequence to selected CtrA homologues. The degree of shading is determined by using software BOXSHADE. The helix–turn–helix DNA-binding motif is boxed with a dashed line. The conserved Asparate residue is indicated with an asterisk above. Amino acid numbers for each CtrA protein are shown on the left. The GenBank accession numbers for sequences used in this alignment are shown in Fig. 2A. (TIFF) [file pone.0066346.s001.tiff]

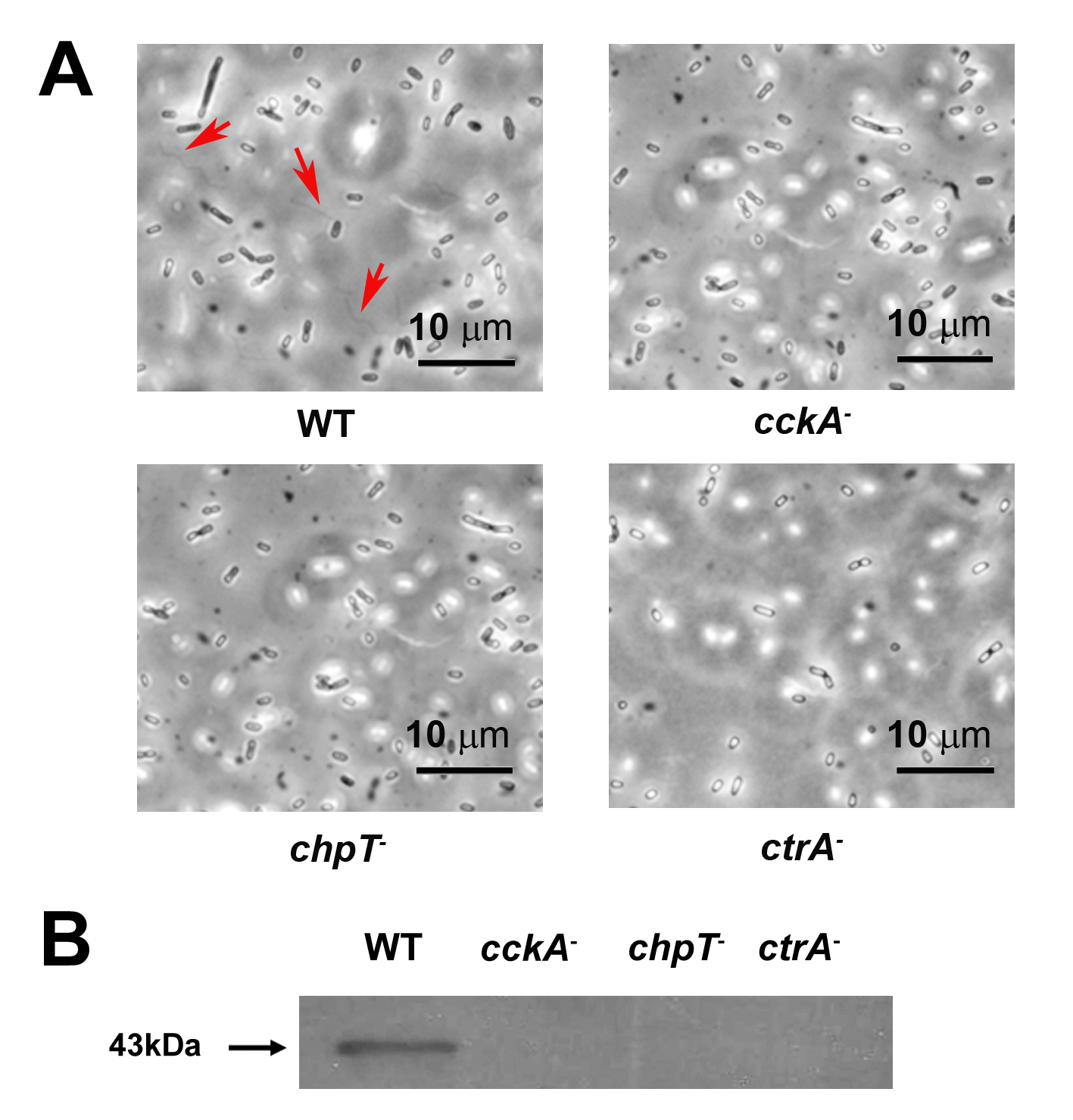

Supplement: Figure S2 — Detection of flagella and flagellin in KLH11 and mutants. A) Flagellar stain of wild type KLH11, cckA −, chpT− and ctrA− null mutants. Stained cells from late stage cultures were viewed under phase contrast microscopy with 100X lens. Wild type (EC1), cckA − (JZ04), chpT − (JZ05), ctrA− (JZ06), Red arrows indicate stained flagella. The bar represents 10 µm. B) Detection of flagellin in wild type KLH11, cckA −, chpT− and ctrA− null mutants. Antibody raised against C. crescentus whole flagella was used to probe for flagellin. Samples were collected at stationary phase. Flagellin was extracted from 3 ml late stage culture from each of the 4 strains with similar OD600. The extraction was dissolved in 100 µl 1X sample buffer and boiled for 5 min. 30 µl was loaded onto each lane. Estimated size of KLH11 flagellin is 43 kDa. (TIFF) [file pone.0066346.s002.tiff]

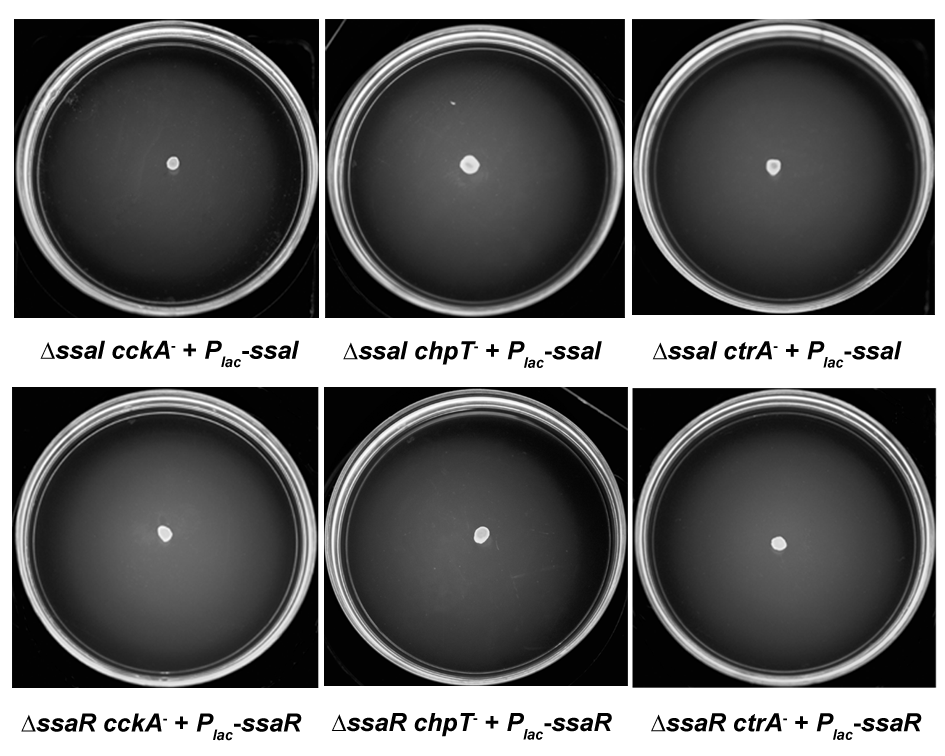

Supplement: Figure S3 — The cckA-chpT-ctrA phosphorely system is required for the ssaRI system to control motility. Plac-ssaI (pEC108) was conjugated into ΔssaI cckA−, ΔssaI cphT− and ΔssaI ctrA − double mutants and Plac-ssaR (pEC112) was conjugated into ΔssaR cckA−, ΔssaR cphT− and ΔssaR ctrA − double mutants, respectively. Strains were inoculated on MB2216 (supplemented with 0.25% agar) swim agar plates for about 8 days at 28°C. The results were representatives of several independent experiments each with three biological replicates. (TIFF) [file pone.0066346.s003.tiff]

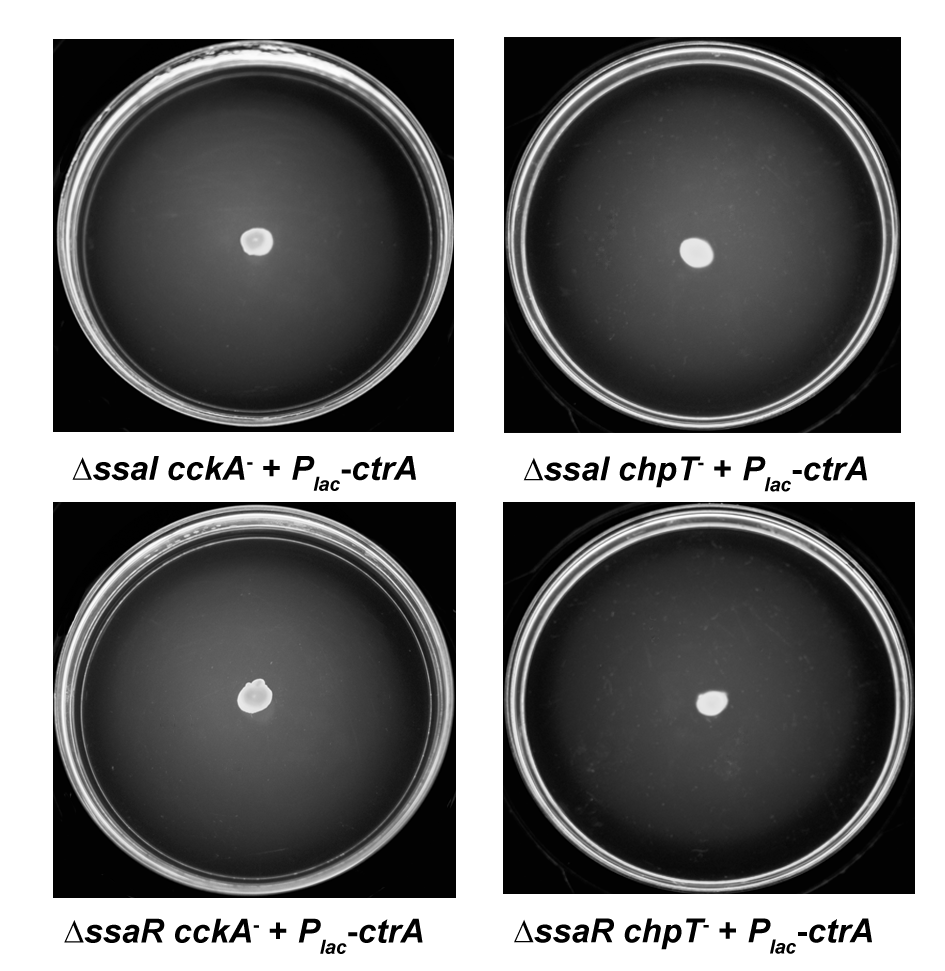

Supplement: Figure S4 — The cckA and chpT genes are required for the function of CtrA. Plac-ctrA was conjugated into ΔssaI cckA−, ΔssaI chpT− , ΔssaR cckA−, and ΔssaR chpT− , respectively. The conjugants were selected and inoculated for swim motility assay as described above. The results were representatives of several independent experiments each with three biological replicates. (TIFF) [file pone.0066346.s004.tiff]
