# Supplementary material for: The CckA-ChpT-CtrA Phosphorelay System Is Regulated by Quorum Sensing and Controls Flagellar Motility in the Marine Sponge Symbiont Ruegeria sp. KLH11
Source: PLoS One. 2013 Jun 25;8(6):e66346. doi: 10.1371/journal.pone.0066346 (PMC3692519; doi:10.1371/journal.pone.0066346)
Supplement: Table S1 — Strains and plasmids used in this study. (DOCX) [file pone.0066346.s005.docx]

**Table S1. Strains and plasmids**

| Bacteria/Plasmid | Relevant feature^a^ | Reference |
| --- | --- | --- |
| *E. coli* TOP 10 F´ | Standard alpha-complementation strain, *lacI^Q^* | Qiagen |
| *E. coli* DH5α/λpir | Strain for propagating R6K suicide plasmids | Lab collection |
| *E. coli* S17-1/λpir | IncP conjugal donor | [[1](#_ENREF_1)] |
| *A. tumefaciens* NTL4 | Ti plasmidless derivative, nopaline  chromosomal background | [[2](#_ENREF_2)] |
| KLH11 | wild type | [[3](#_ENREF_3)] |
| KLH11-EC1 | Rif^R^ | [[4](#_ENREF_4)] |
| KLH11-SK01 | Δ*ssaI,* Rif^R^ | [[4](#_ENREF_4)] |
| KLH11-OKC8 | *fliC::*pOKC12, Km^R^ | [[4](#_ENREF_4)] |
| KLH11-JZ03 | Δ*ssaR,* Rif^R^ | This study |
| KLH11-JZ04 | *cckA*::pJZ003*,* Rif^R^, Km^R^ | This study |
| KLH11-JZ05 | *chpT*::pJZ004*,* Rif^R^, Km^R^ | This study |
| KLH11-JZ06 | *ctrA*::pJZ005*,* Rif^R^, Km^R^ | This study |
| KLH11-JZ07 | Δ*ssaI cckA*::pJZ003*,* Rif^R^, Km^R^ | This study |
| KLH11-JZ08 | Δ*ssaI chpT*::pJZ004*,* Rif^R^, Km^R^ | This study |
| KLH11-JZ09 | Δ*ssaI ctrA*::pJZ005*,* Rif^R^, Km^R^ | This study |
| KLH11-JZ10 | Δ*ssaR cckA*::pJZ003*,* Rif^R^, Km^R^ | This study |
| KLH11-JZ11 | Δ*ssaR chpT*::pJZ004*,* Rif^R^, Km^R^ | This study |
| KLH11-JZ12 | Δ*ssaR ctrA*::pJZ005*,* Rif^R^, Km^R^ | This study |
| KLH11-JZ13 | *cckA*::pJZ012*,*  wild type *cckA*, Rif^R^, Km^R^ | This study |
| pCR 2.1-TOPO^®^ | PCR fragment cloning vector, Ap/Km^R^ | Invitrogen |
| pBBR1-MCS5 | *P_lac_* expression vector, Gm^R^ | [[5](#_ENREF_5)] |
| pNPTS138 | colE1 origin, sacB, Km^R^ | gift of M. Alley |
| pSRKGm | pBBR1MCS-5-derived expression vector containing lac promoter lacI^q^, Gm^R^ | [[6](#_ENREF_6)] |
| pVIK112 | R6K-based *lacZ* transcriptional fusion, integration vector, Km^R^ | [[1](#_ENREF_1)] |
| pRA301 | *lacZ* translational fusion vector | [[7](#_ENREF_7)] |
| pEC108 | pBBR1-MCS5 derivative carrying full length *P_lac_-ssaI*, Gm^R^ | [[4](#_ENREF_4)] |
| pEC112 | pBBR1-MCS5 derivative, carrying full length *P_lac_-ssaR*, Gm^R^ | [[4](#_ENREF_4)] |
| pOKC12 | pVIK112 derivative carrying truncated *fliC*, Km^R^ | Choi and Fuqua, unpublished |
| pJZ003 | pVIK112 derivative carrying truncated *cckA* gene, Km^R^ | This study |
| pJZ004 | pVIK112 derivative carrying truncated *chpT* gene, Km^R^ | This study |
| pJZ005 | pVIK112 derivative carrying truncated *ctrA* gene, Km^R^ | This study |
| pJZ006 | pSRKGm derivative carrying full length *P_lac_-cckA*, Gm^R^ | This study |
| pJZ007 | pSRKGm derivative carrying full length *P_lac_-chpT*, Gm^R^ | This study |
| pJZ008 | pSRKGm derivative carrying full length *P_lac_-ctrA*, Gm^R^ | This study |
| pJZ009 | pRA301 derivative, *P_cckA_-lacZ*, Sp^R^ | This study |
| pJZ010 | pRA301 derivative, *P_chpT_-lacZ*, Sp^R^ | This study |
| pJZ011 | pRA301 derivative, *P_ctrA_-lacZ*, Sp^R^ | This study |
| pJZ012 | pVIK112 derivative, *cckA* gene with 5’  truncation, to retain wt *cckA*, Km^R^ | This study |
| pJZ014 | pNPTS138 carrying *ssaR* deletion fragment, Km^R^ | This study |
| pJEH010 | pSRKGm derivative carrying full length *P_lac_-cckA of A. tumefaciens*, Gm^R^ | [[8](#_ENREF_8)] |
| pJEH027 | pSRKGm derivative carrying full length *P_lac_-chpT of A. tumefaciens*, Gm^R^ | This study |
| pJEH028 | pSRKGm derivative carrying full length *P_lac_-ctrA of A. tumefaciens*, Gm^R^ | This study |

^a^Ap=ampicillin, Gm=gentamicin, Km=kanamycin, Rif=rifampicin, Sp=spectinomycin.

**References**

1. Kalogeraki VS, Winans SC (1997) Suicide plasmids containing promoterless reporter genes can simultaneously disrupt and create fusions to target genes of diverse bacteria. Gene 188: 69-75.

2. Zhu J, Beaber JW, More MI, Fuqua C, Eberhard A, et al. (1998) Analogs of the autoinducer 3-oxooctanoyl-homoserine lactone strongly inhibit activity of the TraR protein of *Agrobacterium tumefaciens*. J Bacteriol 180: 5398-5405.

3. Mohamed NM, Cicirelli EM, Kan J, Chen F, Fuqua C, et al. (2008) Diversity and quorum-sensing signal production of *Proteobacteria* associated with marine sponges. Environ Microbiol 10: 75-86.

4. Zan J, Cicirelli EM, Mohamed NM, Sibhatu H, Kroll S, et al. (2012) A complex LuxR-LuxI type quorum sensing network in a roseobacterial marine sponge symbiont activates flagellar motility and inhibits biofilm formation. Mol Microbiol 85: 916-933.

5. Kovach ME, Elzer PH, Hill DS, Robertson GT, Farris MA, et al. (1995) Four new derivatives of the broad-host-range cloning vector pBBR1MCS, carrying different antibiotic-resistance cassettes. Gene 166: 175-176.

6. Khan SR, Gaines J, Roop II RM, Farrand SK (2008) Broad-host-range expression vectors with tightly regulated promoters and their use to examine the influence of TraR and TraM expression on Ti plasmid quorum sensing. Appl Environ Microbiol 74: 5053-5062.

7. Akakura R, Winans SC (2002) Mutations in the *occQ* operator that decrease OccR-induced DNA bending do not cause constitutive promoter activity. J Biol Chem 277: 15773-15780.

8. Kim J, Heindl JE, Fuqua C (2013) Coordination of division and development influences complex multicellular behavior in *Agrobacterium tumefaciens*. PLoS ONE 8(2): e56682.
