# Supplementary material for: The CckA-ChpT-CtrA Phosphorelay System Is Regulated by Quorum Sensing and Controls Flagellar Motility in the Marine Sponge Symbiont Ruegeria sp. KLH11
Source: PLoS One. 2013 Jun 25;8(6):e66346. doi: 10.1371/journal.pone.0066346 (PMC3692519; doi:10.1371/journal.pone.0066346)
Supplement: Table S2 — Primers used in this study. (DOCX) [file pone.0066346.s006.docx]

**Table S2. Primers used in this study.**

| **Primer name** | **Sequences^a^ (5’-3’)** | **Restriction enzyme** |
| --- | --- | --- |
| cckA P1 | GAATTCCGCGCAAGGACAAAGAGATA | *Eco*RI |
| cckA P2 | GGTACCCGACAAGGTTCATCAACACC | *Kpn*I |
| cckA P3 | CCGCTCTAGAACTAGTGTGAAAC*AGGAAA*CAGCT**ATG**TCCAGTGTTTCTGAATC | *Spe*I |
| cckA P4 | CGGGGGATCCACTAGT**CTA**GTTGAGTTGCTGGAAC | *Spe*I |
| cckA P5 | GAATTCCGGAACCGATGGATTTTACA | *EcoR*I |
| cckA P6 | CTGCAGCGACACATAGCGGCCGTGGTC | *Pst*I |
| chpT P1 | GAATTCCGCATCAGACGTCAACCTT | *Eco*RI |
| chpT P2 | GGTACCCTTTCCAGAGGCCGCTATC | *Kpn*I |
| chpT P3 | CCGCTCTAGAACTAGTG**TGA**AAC*AGGAAA*CAGCT**ATG**CAGCAGGAGGTACGCATG | *Spe*I |
| chpT P4 | CGGGGGATCCACTAGT**CTA**AAAGCGCAGCGTTACAC | *Spe*I |
| chpT P5 | GAATTCGGATCAAAGACCGGCATCAG | *Eco*RI |
| chpT P6 | CTGCAGCTG**CAT**CTGCCGCAGGAGCCG | *Pst*I |
| ctrA P1 | GAATTCGATGCTGACACATGCCAATC | *Eco*RI |
| ctrA P2 | GGTACCATCTCTTTCGTCAGCGTGGT | *Kpn*I |
| ctrA P3 | CCGCTCTAGAACTAGTG**TGA**AAC*AGGAAA*CAGCT**ATG**CGAATACTTCTCGTCGA | *Spe*I |
| ctrA P4 | CGGGGGATCCACTAGT**TCA**GGCGCCGACCGCCA | *Spe*I |
| ctrA P5 | GAATTCGGCGGAACATGGCGTCGA | *Eco*RI |
| ctrA P6 | CTGCAGTCG**CAT**TCAACTGCTCCAAT | *Pst*I |
| ssaR D1 | CTGGATCCACGAATTCGGTAAACCGCCCCTATTACGG | *Eco*RI |
| ssaR D2 | **AAGCTTGGTACCGAATTC**AATATC**CAT**CGGTAACGACCA | NA |
| ssaR D3 | **GAATTCGGTACCAAGCTT**CCAGGT**TAA**AACCAAAACTCC | NA |
| ssaR D4 | CGAAGCTAGCGAATTCGTCGCATAGGACACCGAGTTC | *Eco*RI |
| JEH48 | gaagaaCAT**ATG**ACGAGCAAACTCAATATCACGC | *Nde*I |
| JEH53 | gaagaaGCTAGC**TCA**CTCGGCAACCGTCTTTGC | *Nhe*I |
| JEH50 | gaagaaCAT**ATG**CGGGTTCTACTGATTGAAGACGA | *Nde*I |
| JEH54 | gaagaaGCTAGC**TCA**GGCGGTTTCGAGGAA | *Nhe*I |
| rpoDRT1 | GACGCCTATCGCGGCCGT | NA |
| rpoDRT2 | GCCGACCTGCGCCATATCGT | NA |
| RT-FliFF | GCGCGGTGTTGCCTATGAGAT | NA |
| RT-FliFR | GATGCCGCGAATCGCTGG T | NA |
| RT-FlhAF | CGGGCTTCTGATCACGCTCCT | NA |
| RT-FlhAR | GCGTTGTCAGTGGCACCTTGT | NA |
| RT-FlgBF | TACGCAATGGCAACCCATGCT | NA |
| RT-FlgBR | GCGTCCGAAATGCCATGCAGAT | NA |
| RT-motBF | GTGACGGCCATGATGGCGTT | NA |
| RT-motBR | CCTTGTGCATCCACGCCTGT | NA |
| RT-FlgJF | GCTTTGGTTCCACAACAGCTAA | NA |
| RT-FlgJR | CTGTTGCTGCACGAGGAAAG | NA |
| fliCRT1 | CGCAGAACCTGTCGACCGGT | NA |
| fliCRT2 | GATGCCGCGAATCGCTGGT | NA |
| RT-ftsZF | GCAGCTGGACGGCGTTGAAT | NA |
| RT-ftsZR | CCGCCAGATGATCCACGATCTGT | NA |
| RT-ccrMF | GTCGACGCGGTCGATGATCACT | NA |
| RT-ccrMR | GTTCGACTTGCGCCACACAACAT | NA |
| cckAintactF | GAATTCACAGGTCTGGGTCTGTCCAC | *Eco*RI |
| cckAintactR | GGTACC**CTA**GTTGAGTTGCTGGAAC | *Kpn*I |
| 112R | GGCTGCAGGTCGACCATGGTC | NA |

^a^ Engineered restriction sequences are underlined. Complementary sequences for PCR-SOEing are shown in bold and are also underlined. Start and stop codons are in bold. *E. coli* *lacZ* ribosomal binding sites are in italics. Protection nucleotides are in lower case. NA= not applicable.
