# Supplementary material for: The CckA-ChpT-CtrA Phosphorelay System Is Regulated by Quorum Sensing and Controls Flagellar Motility in the Marine Sponge Symbiont Ruegeria sp. KLH11
Source: PLoS One. 2013 Jun 25;8(6):e66346. doi: 10.1371/journal.pone.0066346 (PMC3692519; doi:10.1371/journal.pone.0066346)
Supplement: Table S3 — Quantification of ftsZ and ccrM expression by qRT-PCR. (DOCX) [file pone.0066346.s007.docx]

**Table S3**. **Quantification of *ftsZ* and *ccrM* expression by qRT-PCR.**

| Gene name | Wild-type ^a^ | *ctrA*^- a^ |
| --- | --- | --- |
| *ftsZ* | 9.9 (3.9) | 10.9 (3.2) |
| *ccrM* | 1.3 (0.4) | 1.5 (0.3) |

^a^Value relative to the housekeeping gene *rpoD.*  Mean (standard deviation). The results presented are representative of two independent experiments each with three biological replicates.
