# Supplementary material for: The CckA-ChpT-CtrA Phosphorelay System Is Regulated by Quorum Sensing and Controls Flagellar Motility in the Marine Sponge Symbiont Ruegeria sp. KLH11
Source: PLoS One. 2013 Jun 25;8(6):e66346. doi: 10.1371/journal.pone.0066346 (PMC3692519; doi:10.1371/journal.pone.0066346)
Supplement: Table S4 — Regulation of cckA by ctrA . (DOCX) [file pone.0066346.s008.docx]

**Table S4. Regulation of *cckA* by *ctrA.***

**Strain genotype Expression plasmid β-Gal. Sp. Act.^1^**

*cckA-lacZ,* WT *cckA* (JZ13) Vector (pSRKGm) 14.5 (0.9)

*cckA-lacZ,* WT *cckA* (JZ13) *P_lac_-ctrA* (pJZ008) 13.0 (0.1)

^1^ Specific activity in Miller units, averages of assays in triplicate (standard deviation) and representative results of two independent experiments each with three biological replicate. IPTG concentration was 200 µM.
