# Supplementary material for: The CckA-ChpT-CtrA Phosphorelay System Is Regulated by Quorum Sensing and Controls Flagellar Motility in the Marine Sponge Symbiont Ruegeria sp. KLH11
Source: PLoS One. 2013 Jun 25;8(6):e66346. doi: 10.1371/journal.pone.0066346 (PMC3692519; doi:10.1371/journal.pone.0066346)
Supplement: Table S5 — Expression of KLH11 PcckA, PchpT and PctrA promoters in an AHL− host. (DOCX) [file pone.0066346.s009.docx]

**Table S5. Expression of KLH11 *P_cckA_, P_chpT_* and *P_ctrA_* promoters in an AHL^-^ host^1^**

**β-Gal Sp. Act.^2^**

**Expression plasmid Fusion No AHL + AHL^3^**

Vector (pBBR1-MCS5) *cckA-lacZ* (pJZ009) 299.4 (38.0) 326.9 (33.4)

*P_lac_-ssaR* (pEC112) *cckA-lacZ* (pJZ009) 295.0 (26.6) 328.7 (12.4)

Vector (pBBR1-MCS5) *chpT-lacZ* (pJZ010) 33.4 (1.2) 32.5 (1.2)

*P_lac_-ssaR* (pEC112) *chpT-lacZ* (pJZ010) 24.3 (0.5) 23.3 (1.9)

Vector (pBBR1-MCS5) *ctrA-lacZ* (pJZ011) 123.8 (20.2) 139.4 (2.8)

*P_lac_-ssaR* (pEC112) *ctrA-lacZ* (pJZ011) 138.6 (18.6) 137.1 (10.1)

^1^ All strains derived from Ti-plasmidless *A. tumefaciens* NTL4.

^2^Specific activity in Miller units, averages of assays in triplicate (standard deviation) and representative results of two independent experiments each with three biological replicates.

^3^ 3-oxo-C16:1 ∆11-HSL (2 µM ) was added.
